# Growth and CD4 patterns of adolescents living with perinatally acquired HIV worldwide, a CIPHER cohort collaboration analysis

**DOI:** 10.1002/jia2.25871

**Published:** 2022-03-07

**Authors:** The Collaborative Initiative for Paediatric HIV Education and Research (CIPHER) Global Cohort Collaboration, Julie Jesson, Siobhan Crichton, Matteo Quartagno, Marcel Yotebieng, Elaine J. Abrams, Kulkanya Chokephaibulkit, Sophie Le Coeur, Marie‐Hélène Aké‐Assi, Kunjal Patel, Jorge Pinto, Mary Paul, Rachel Vreeman, Mary‐Ann Davies, Jihane Ben‐Farhat, Russell Van Dyke, Ali Judd, Lynne Mofenson, Marissa Vicari, George Seage, Linda‐Gail Bekker, Shaffiq Essajee, Diana Gibb, Martina Penazzato, Intira Jeannie Collins, Kara Wools‐Kaloustian, Amy Slogrove, Kate Powis, Paige Williams, Mogomotsi Matshaba, Lineo Thahane, Phoebe Nyasulu, Bhekumusa Lukhele, Lumumba Mwita, Adeodata Kekitiinwa‐Rukyalekere, Sebastian Wanless, Tessa Goetghebuer, Claire Thorne, Josiane Warszawski, Luisa Galli, Annemarie M.C van Rossum, Carlo Giaquinto, Magdalena Marczynska, Laura Marques, Filipa Prata, Luminita Ene, Lyuba Okhonskaya, Marisa Navarro, Antoinette Frick, Lars Naver, Christian Kahlert, Alla Volokha, Elizabeth Chappell, Jean William Pape, Vanessa Rouzier, Adias Marcelin, Regina Succi, Annette H. Sohn, Azar Kariminia, Andrew Edmonds, Patricia Lelo, Rita Lyamuya, Edith Apondi Ogalo, Francesca Akoth Odhiambo, Andreas D. Haas, Carolyn Bolton, Josephine Muhairwe, Hannock Tweya, Mariam Sylla, Marceline D'Almeida, Lorna Renner, Mark J. Abzug, James Oleske, Murli Purswani, Chloe Teasdale, Harriet Nuwagaba‐Biribonwoha, Ruth Goodall, Valériane Leroy

**Affiliations:** ^1^ CERPOP Inserm Université Paul Sabatier Toulouse 3 Toulouse France; ^2^ MRC Clinical Trials Unit University College London London UK; ^3^ Division of General Internal Medicine Department of Medicine Albert Einstein College of Medicine Bronx New York USA; ^4^ ICAP at Columbia University Mailman School of Public Health Columbia University New York USA; ^5^ Siriraj Institute of Clinical Research Faculty of Medicine Siriraj Hospital Mahidol University Salaya Thailand; ^6^ Institut National d'Etude Demographique (INED) Mortality, Health and Epidemiology Unit Paris France; ^7^ Institut de Recherche pour le Developpement (IRD) UMI‐174/PHPT Faculty of Associated Medical Sciences Chiang Mai University Chiang Mai Thailand; ^8^ University Hospital Yopougon Abidjan Côte d'Ivoire; ^9^ Harvard T. H. Chan School of Public Health Boston Massachusetts USA; ^10^ Department of Pediatrics School of Medicine Federal University of Minas Gerais Belo Horizonte Brazil; ^11^ Baylor International Pediatric AIDS Initiative Texas Children's Hospital‐USA Houston Texas USA; ^12^ Department of Global Health Icahn School of Medicine at Mount Sinai New York USA; ^13^ School of Public Health and Family Medicine Faculty of Health Sciences University of Cape Town Cape Town South Africa; ^14^ Epicentre Médecins Sans Frontières Paris France; ^15^ Tulane University Health Sciences Center New Orleans Louisiana USA; ^16^ Elizabeth Glaser Pediatric AIDS Foundation Washington DC USA; ^17^ International AIDS Society Geneva Switzerland; ^18^ Desmond Tutu HIV Centre University of Cape Town Cape Town South Africa; ^19^ UNICEF New York USA; ^20^ HIV Department World Health Organization Geneva Switzerland; ^21^ Indiana University School of Medicine Indianapolis Indiana USA; ^22^ Department of Paediatrics & Child Health Faculty of Medicine & Health Sciences Stellenbosch University Worcester South Africa; ^23^ Baylor College of Medicine Children's Foundation Lilongwe Botswana; ^24^ Baylor College of Medicine Children's Foundation Maseru Lesotho; ^25^ Baylor College of Medicine Children's Foundation Lilongwe Malawi; ^26^ Baylor College of Medicine Children's Foundation Mbabane, eSwatini; ^27^ Baylor College of Medicine Children's Foundation Mwanza Tanzania; ^28^ Baylor College of Medicine Children's Foundation Kampala Uganda; ^29^ Hospital St Pierre Brussels Belgium; ^30^ UCL Great Ormond Street Institute of Child Health University College London London UK; ^31^ Inserm U1018 Centre de recherche en Epidémiologie et Santé des Populations Paris France; ^32^ Infection Disease Unit Meyer Children's University Hospital Florence Italy; ^33^ Department of Health Sciences University of Florence Florence Italy; ^34^ Erasmus MC University Medical Center Rotterdam‐Sophia Children's Hospital Rotterdam The Netherlands; ^35^ Padova University/PENTA Foundation Padua Italy; ^36^ Medical University of Warsaw Hospital of Infectious Diseases in Warsaw Warsaw Poland; ^37^ Centro Hospitalar do Porto Porto Portugal; ^38^ Hospital de Santa Maria Lisboa Portugal; ^39^ Victor Babes Hospital Bucharest Romania; ^40^ Republican Hospital of Infectious Diseases St Petersburg Russian Federation; ^41^ Hospital General Universitario “Gregorio Marañón” Madrid Spain; ^42^ Hospital Universitari Vall d' Hebron Vall d' Hebron Research Institute Universitat Autònoma de Barcelona Barcelona Spain; ^43^ Karolinska University Hospital and Karolinska Institutet Stockholm Sweden; ^44^ Children's Hospital of Eastern Switzerland Saint Gallen Switzerland; ^45^ Shupyk National Medical Academy of Postgraduate Education Kiev Ukraine; ^46^ GHESKIO Center Port‐au‐Prince Haiti; ^47^ Universidade Federal de Sao Paulo Sao Paulo Brazil; ^48^ TREAT Asia/amfAR Bangkok Thailand; ^49^ Kirby Institute University of New South Wales Sydney New South Wales Australia; ^50^ Gillings School of Global Public Health University of North Carolina at Chapel Hill Chapel Hill North Carolina USA; ^51^ Pediatric Hospital Kalembe Lembe Lingwala, Demogratic Republic of Congo; ^52^ Morogoro Regional Hospital Morogoro Tanzania; ^53^ Moi Teaching and Referral Hospital Eldoret Kenya; ^54^ Center for Microbiology Research Kenya Medical Research Institute Nairobi Kenya; ^55^ Institute of Social and Preventive Medicine University of Bern Bern Switzerland; ^56^ Centre for Infectious Disease Research in Zambia Lusaka Zambia; ^57^ SolidarMed Lesotho Zimbabwe; ^58^ Lighthouse Trust Clinic Lilongwe Malawi; ^59^ CHU Gabriel Toure Bamako Mali; ^60^ Centre National Hospitalier Universitaire Hubert K. Maga Cotonou Benin; ^61^ Korle Bu Teaching Hospital Accra Ghana; ^62^ University of Colorado School of Medicine and Children's Hospital Colorado Aurora Colorado USA; ^63^ Rutgers ‐ New Jersey Medical School Newark New Jersey USA; ^64^ Bronx‐Lebanon Hospital Center Bronx New York USA

**Keywords:** adolescent, CD4, cohort studies, growth, HIV, perinatally acquired

## Abstract

**Introduction:**

Adolescents living with HIV are subject to multiple co‐morbidities, including growth retardation and immunodeficiency. We describe growth and CD4 evolution during adolescence using data from the Collaborative Initiative for Paediatric HIV Education and Research (CIPHER) global project.

**Methods:**

Data were collected between 1994 and 2015 from 11 CIPHER networks worldwide. Adolescents with perinatally acquired HIV infection (APH) who initiated antiretroviral therapy (ART) before age 10 years, with at least one height or CD4 count measurement while aged 10–17 years, were included. Growth was measured using height‐for‐age Z‐scores (HAZ, stunting if <‐2 SD, WHO growth charts). Linear mixed‐effects models were used to study the evolution of each outcome between ages 10 and 17. For growth, sex‐specific models with fractional polynomials were used to model non‐linear relationships for age at ART initiation, HAZ at age 10 and time, defined as current age from 10 to 17 years of age.

**Results:**

A total of 20,939 and 19,557 APH were included for the growth and CD4 analyses, respectively. Half were females, two‐thirds lived in East and Southern Africa, and median age at ART initiation ranged from <3 years in North America and Europe to >7 years in sub‐Saharan African regions. At age 10, stunting ranged from 6% in North America and Europe to 39% in the Asia‐Pacific; 19% overall had CD4 counts <500 cells/mm^3^. Across adolescence, higher HAZ was observed in females and among those in high‐income countries. APH with stunting at age 10 and those with late ART initiation (after age 5) had the largest HAZ gains during adolescence, but these gains were insufficient to catch‐up with non‐stunted, early ART‐treated adolescents. From age 10 to 16 years, mean CD4 counts declined from 768 to 607 cells/mm^3^. This decline was observed across all regions, in males and females.

**Conclusions:**

Growth patterns during adolescence differed substantially by sex and region, while CD4 patterns were similar, with an observed CD4 decline that needs further investigation. Early diagnosis and timely initiation of treatment in early childhood to prevent growth retardation and immunodeficiency are critical to improving APH growth and CD4 outcomes by the time they reach adulthood.

## 1 INTRODUCTION

In 2019, an estimated 1.7 million adolescents aged 10–19 years were living with HIV worldwide, with 90% of them in sub‐Saharan Africa, and 8% in Asia and the Pacific [1]. They either acquired HIV during adolescence or are adolescents living with perinatally acquired HIV (APH). APH experience specific challenges due to their life‐long infection and long‐term follow‐up in HIV care [2, 3, 4, 5, 6]. They are at risk of inadequate adherence to antiretroviral therapy (ART) [7, 8] and poor retention in care during adolescence [9, 10]. APH may in turn experience poor health outcomes [11], with a high mortality rate [12, 13], virologic failure [14, 15] and HIV drug resistance [16], poor growth [17] and immune status [18].

Previous work has demonstrated that linear growth improves after ART initiation, with better outcomes associated with earlier ART [19, 20], but long‐term growth evolution on ART has been less described [21, 22], especially in APH [17, 23]. Some studies have described APH growth velocity in resource‐limited settings [17] and in Europe and Thailand [23], showing lower height gains and a delayed growth spurt for APH compared to HIV‐uninfected adolescents.

Similarly, the immune response following ART initiation has been extensively described in children [24, 25] and adults [26, 27] living with HIV, but long‐term CD4 evolution on ART for APH has not been sufficiently explored. In previous studies looking at patterns of long‐term trends in CD4 count in children living with HIV in sub‐Saharan Africa [28] and Europe [29], CD4 counts at adolescence were hypothesized to remain constant after the initial CD4 response following ART initiation, likely reaching a similar level to that of the non‐HIV‐infected population of the same age [28].

Our primary objective was to describe linear growth and immune evolution across adolescence by sex and geographical region among ART‐treated APH enrolled in the Collaborative Initiative for Paediatric HIV Education and Research (CIPHER) global project. A secondary objective was to explore the correlation between growth and CD4 evolution.

## 2 METHODS

### 2.1 Study population

This multiregional study was based on pooled data from the CIPHER collaboration. This global network of observational paediatric HIV cohorts previously described the epidemiology of adolescents living with HIV worldwide [30], with a focus in sub‐Saharan Africa [31]. For this study, the following 11 cohort networks contributed data: Baylor International Pediatric AIDS Initiative (BIPAI); European Pregnancy and Paediatric Infections Cohort Collaboration (EPPICC); the International Epidemiology Databases to Evaluate AIDS (IeDEA) Consortium (IeDEA Asia‐Pacific, IeDEA Central Africa, IeDEA East Africa, IeDEA Southern Africa, IeDEA West Africa, the Caribbean, Central and South America network (CCASAnet)); International Maternal Pediatric Adolescent AIDS Clinical Trials (IMPAACT) 219C and P1074; Optimal Models (ICAP at Columbia University); and Pediatric HIV/AIDS Cohort Study (PHACS). Pooled individual participant‐level data were thus collected from high‐, middle‐ and low‐income countries and drawn from a range of care settings, including dedicated research cohorts, routine care cohorts and programmatic services.

The inclusion criteria were: adolescents aged 10–19 years, documented perinatally acquired HIV or unknown mode of infection and aged<10 years at first presentation to HIV care (proxy perinatal HIV transmission), age <10 years at ART initiation and ≥1 height or CD4 count measurement between age 10 and 19 years to contribute to the growth and CD4 analyses, respectively. The analysis was subsequently truncated to include only measurements from age 10 to 17 years due to small sample size at 18 and 19 years of age.

Each participating cohort in CIPHER obtained ethics approval from their respective institutional review boards for data collection and transfer. Consent or assent requirements for study participation were deferred to local institutional review boards. All analyses were pre‐specified and agreed by the CIPHER Adolescent Study Group.

### 2.2 Outcomes and exploratory variables

Two main outcomes of interest were investigated: (1) growth, defined by height‐for‐age Z‐score (HAZ), based on the WHO Child Growth standards [32, 33] and (2) CD4 counts (cells/mm^3^). CD4 counts were also expressed as an age‐adjusted CD4 ratio, obtained by dividing observed CD4 counts by CD4 reference values in HIV‐uninfected adolescents of the same age [34]. The prevalence of malnutrition was assessed by stunting (i.e. HAZ <‐2 Standard Deviations [SD]) and wasting (i.e. weight‐for‐height Z‐score [WHZ] <‐2 SD if <5 years old, BMI‐for‐age Z‐score [BAZ] <‐2 SD if older), severe malnutrition was defined as Z‐scores <‐3 SD. Immunodeficiency at ART initiation was determined according to the 2006 WHO guidelines [35], immunodeficiency at age 10 was defined as severe (CD4 counts <250/mm^3^), moderate (250–500/mm^3^) and not immunodeficient (>500/mm^3^). Regions were combined as follows: North America and Europe (NA&E); Central, South America and the Caribbean (CSA&C); Asia‐Pacific; West and Central Africa (West&CA); Botswana and South Africa (Botsw.&SA); East and Southern Africa (East&SA) (excluding Botswana and South Africa). Botswana and South Africa, both upper middle‐income countries, were classified as a separate group within sub‐Saharan Africa. Adolescents were defined as alive and retained in care, transferred out, dead or lost‐to‐follow‐up if no visit were observed for >365 days after their last observed visit.

### 2.3 Statistical analyses

For both study populations contributing to the growth and CD4 analyses, characteristics were described at ART initiation and at age 10, and compared by sex and geographical region, using Chi‐square tests for categorical variables and Kruskal–Wallis tests for continuous variables. We compared characteristics of the study population and adolescents who were excluded, due to lack of data on height or CD4 measurement and because they did not initiate ART by age 10 years.

Growth and CD4 evolution were described between age 10 and 17 years by plotting mean HAZ and CD4 count over time, by sex and regions. Growth and CD4 modelling included the following parameters: age at ART initiation, immunodeficiency and stunting at ART initiation and at age 10, wasting at age 10 (growth modelling only) and year of birth (CD4 modelling only), regions and sex. For the growth model, non‐linearity of some parameters and the heterogeneity of results by sex and regions had to be taken into account. Growth analyses were thus stratified by sex and included fractional polynomials [36] for the following continuous variables: age at ART initiation, HAZ at age 10 years and time, defined as current age from 10 to 17 years of age. The fractional polynomial terms were inserted into a linear mixed model, with an exponential spatial correlation structure, and interaction between time and the explanatory variables. For the CD4 evolution modelling, a linear mixed model with an unstructured variance‐covariance matrix and interaction with time was fitted. Variables were selected in univariate models if *p*<0.25 and kept in final multivariate models if *p*<0.05 using a backward selection method.

A secondary objective was to explore the possible correlation between HAZ and CD4 evolution among APH, implementing multivariate multilevel models [37] and a multi‐trajectory analysis [38]. The first strategy consisted of fitting a bivariate normal joint model estimating the correlation between the two outcomes at a specific time and the correlation between the two outcomes evolution over time. The second strategy's aim was to identify profiles of co‐evolution of CD4 and HAZ.

Analyses were conducted using SAS version 9.3 (SAS Institute Inc., Cary, NC, USA), fractional polynomials were initially determined using Stata version 13.0 (StataCorp, College Station, TX, USA) and the “fp” package. Figures were plotted using the ggplot2 package in R version 3.2.2 (R Foundation for Statistical Computing, Vienna, Austria).

## 3 RESULTS

### 3.1 Selection and characteristics of the study population

**Figure 1:**
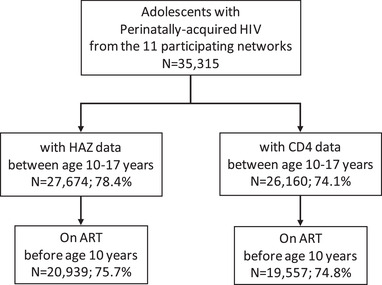
Selection of the adolescents living with HIV for the growth and CD4 analyses. CIPHER global cohort collaboration, 1994–2015.

Between 1994 and 2015, data on 35,315 APH across 46 countries were collected. Respectively, 78% and 74% had ≥ 1 measurement for height and CD4 during adolescence. After restricting to those initiated on ART before age 10 years, 20,939 (76%) APH were included in the growth analyses and 19,557 (75%) in the CD4 analyses (Figure 1).

Characteristics of both study populations are shown in Table 1 by sex and in Tables S1 and S2 by regions. If not specified, results detailed in the text refer to the growth analysis study population, the CD4 analysis study population was broadly similar. Overall, half were females, and two‐thirds lived in East&SA. Median age at ART initiation varied widely by region, ranging from 2.4 years (IQR 0.6;5.7) in NA&E to 7 years and more in sub‐Saharan African regions. At ART initiation, the prevalence of stunting ranged from 8% in NA&E to 40% in Asia‐Pacific, severe immunodeficiency ranged from 20% in NA&E to 50% in the Asia‐Pacific. Half of APH had missing data regarding anthropometric parameters or CD4 measurements at ART initiation. At age 10, the prevalence of stunting ranged from 6% in NA&E to 39% in the Asia‐Pacific. In the CD4 analysis study population, 19% were moderately or severely immunodeficient at age 10 (Table 1).

**Table 1:** Characteristics of study participants for the growth and CD4 analyses, stratified by sex

	Growth analyses							CD4 analyses						
	Total		Males		Females			Total		Males		Females		
Characteristics	(*N* = 20,939)		(*n* = 10,512)		(*n* = 10,427)		*p*‐value*	(*N* = 19,557)		(*n* = 9850)		(*n* = 9707)		*p*‐value*
At ART initiation														
Age, median (IQR)	6.9 (4.4; 8.5)		6.8 (4.4; 8.5)		6.9 (4.4; 8.5)		0.089	6.9 (4.4; 8.5)		6.8 (4.4; 8.5)		6.9 (4.3; 8.5)		0.227
Age groups (years), *n* %							0.017							0.014
0–2	2391	11	1162	11	1229	12		2379	12	1141	12	1238	13	
2–5	3795	18	1977	19	1818	17		3477	18	1802	18	1675	17	
5–10	14,753	70	7373	70	7380	71		13,701	70	6907	70	6794	70	
HAZ, median (IQR)	–1.94 (–2.87; –1.02)		–1.96 (–2.91; –1.06)		–1.92 (–2.83; –1.00)		0.052	–1.92 (–2.84; –1.01)		–1.94 (–2.88; –1.05)		–1.90 (–2.80; –0.97)		0.038
Severity of stunting, *n* %							0.100/0.147							0.225/0.152
None	5785	28	2836	27	2949	28		4755	24	2345	24	2410	25	
Moderate (]–3; –2] SD)	2856	14	1417	13	1439	14		2338	12	1170	12	1168	12	
Severe (<‐3 SD)	2478	12	1273	12	1205	12		1947	10	1011	10	936	10	
Missing	9820	47	4986	47	4834	46		10,517	54	5324	54	5193	53	
WHZ/BAZ, median (IQR)	–0.51 (–1.36; 0.27)		–0.53 (–1.44; 0.32)		–0.50 (–1.29; 0.22)		0.475	–0.51 (–1.36; 0.27)		–0.54 (–1.44; 0.30)		–0.48 (–1.28; 0.24)		0.110
Severity of wasting, *n* %							<0.001							<0.001
None	9492	45	4653	44	4839	46		7717	39	3802	39	3915	40	
Moderate (]–3; –2] SD)	959	5	512	5	447	4		775	4	424	4	351	4	
Severe (<‐3 SD)	564	3	319	3	245	2		462	2	267	3	195	2	
Missing	9924	47	5028	48	4896	47		10,603	54	5357	54	5246	54	
Immunodeficiency by age^a^, *n* %							0.002/0.001							0.020/0.014
None	2940	14	1384	13	1556	15		3093	16	1479	15	1614	17	
Moderate	2090	10	1040	10	1050	10		2209	11	1111	11	1098	11	
Severe	6011	29	3078	29	2933	28		6395	33	3262	33	3133	32	
Missing	9898	47	5010	48	4888	47		7860	40	3998	41	3862	40	
At 10 years of age														
HAZ, median (IQR)	–1.48 (–2.32; –0.67)		–1.41 (–2.21; –0.65)		–1.55 (–2.40; –0.68)		<0.001	–1.38 (–2.22; –0.53)		–1.30 (–2.14; –0.49)		–1.46 (–2.32; –0.57)		<0.001
Severity of stunting, *n* %							<0.001							<0.001
No	11,299	54	5885	56	5414	52		7287	37	3782	38	3505	36	
Moderate (]–3; –2] SD)	4061	19	1992	19	2069	20		2374	12	1164	12	1210	12	
Severe (<‐3 SD)	1755	8	726	7	1029	10		975	5	406	4	569	6	
Missing	3824	18	1909	18	1915	18		8921	46	4498	46	4423	46	
BAZ, median (IQR)	–0.50 (–1.20; 0.18)		–0.49 (–1.22; 0.20)		–0.51 (–1.18; 0.17)		0.810	–0.44 (–1.15; 0.26)		–0.44 (–1.18; 0.27)		–0.44 (–1.11; 0.24)		0.566
Severity of wasting, *n* %							0.012/0.004							0.029/0.011
None	15,455	74	7721	73	7734	74		9708	50	4852	49	4856	50	
Moderate (]–3; –2] SD)	1092	5	563	5	529	5		617	3	326	3	291	3	
Severe (<‐3 SD)	341	2	200	2	141	1		174	1	105	1	69	1	
Missing	4051	19	2028	19	2023	19		9058	46	4567	46	4491	46	
CD4 count, median (IQR)	729 (478; 1003)		705 (466; 978)		753 (490; 1036)		<0.001	731 (491; 998)		710 (477; 507)		751 (507; 1025)		<0.001
Immunodeficiency, *n* %							0.016/0.009							<0.001
None	8919	43	4428	42	4491	43		10,788	55	5305	54	5483	56	
Moderate	1492	7	781	7	711	7		1810	9	964	10	846	9	
Severe	1817	9	964	9	853	8		1921	10	1016	10	905	9	
Missing	8711	42	4339	41	4372	42		5038	26	2565	26	2473	25	
At last visit														
Age, median (IQR)	12.8 (11.3; 14.9)		12.8 (11.3; 14.9)		12.8 (11.3; 14.9)		0.437	13.0 (11.6; 15.0)		13.0 (11.6; 14.9)		13.0 (11.6; 15.0)		0.397
Follow‐up status							0.678							0.857
Alive, in follow‐up	13,803	66	6946	66	6857	66		12,387	63	6246	63	6141	63	
Lost‐to‐follow‐up^b^	3783	18	1878	18	1905	18		4309	22	2154	22	2154	22	
Transferred	3022	14	1513	14	1509	14		2538	13	1281	13	1281	13	
Dead	331	2	178	2	156	2		323	2	169	2	169	2	

Note: Missing categories for stunting, wasting and immunodeficiency correspond to missing data for HAZ, WAZ or CD4 count, respectively.

Abbreviations: ART, antiretroviral therapy; BAZ, BMI‐for‐age Z‐score; ; HAZ, height‐for‐age Z‐score; IQR, interquartile range; WHZ, weight‐for‐height Z‐score.

^a^ By WHO 2006 guidelines.

^b^ Defined as no contact for more than 365 days or documented loss‐to‐follow‐up.

* Chi‐square or Kruskal–Wallis tests, second *p*‐value corresponding to the test excluding the missing category, if different from the initial test.

CIPHER global cohort collaboration, 1994–2015.

Regions differed by follow‐up length, year of birth and data contribution over time. Median age at last visit was 15.2 years (IQR 12.8;17.4) in NA&E versus 12.4 (IQR 11.1;14.1) in West&CA. Mortality rate ranged from 1% to 4% (CSA&C). At last visit, from 9% to 13% were considered lost‐to‐follow‐up in all regions except NA&E where this rate was 31%. Transfer to adult care differed greatly by regions as well, from 2% reported in West&CA to 19% (NA&E) and 21% in Botsw.&SA. Between age 10 and 14 years, East&SA contributed the most growth and CD4 data (72% and 48%, respectively). Between age 15 and 17 years, East&SA data contribution decreased due to shorter follow‐up, with NA&E contributing the majority of CD4 data (60%) (Tables S1 and S2).

Compared to the study population, APH who were excluded because of lack of height or CD4 measurement were more often from sub‐Saharan Africa (87% vs. 73%) and more likely to have missing HAZ or CD4 (70% vs. 47%) at ART initiation. Those excluded because they had initiated ART after age 10 were almost all living in sub‐Saharan Africa (97%). Those who were not part of the growth model because of missing HAZ at age 10 had similar characteristics to those in the analysis.

### 3.2 Growth evolution during adolescence

**Figure 2:**
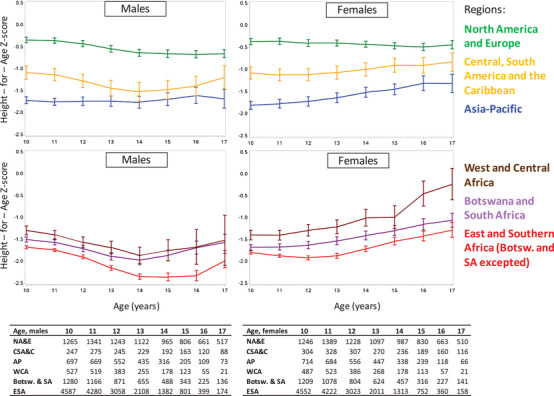
Mean height‐for‐age Z‐score evolution between 10 and 17 years of age, stratified by regions (sub‐Saharan Africa at the bottom and other regions at the top) and sex, with sample size by year. CIPHER global cohort collaboration, 1994–2015.

APH from NA&E had the highest HAZ values throughout adolescence, followed by the Asia‐Pacific and CSA&C. Growth patterns in sub‐Saharan African regions greatly differed by sex, with females experiencing HAZ gains over time, while males experienced a decline in HAZ in early adolescence before a return to HAZ values that were similar by age 17 to those at age 10 years (Figure 2).

**Figure 3:**
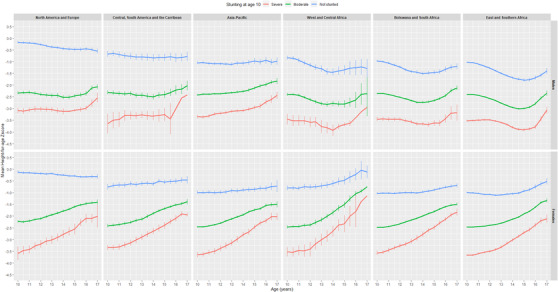
Adjusted estimated mean height‐for‐age Z‐scores for adolescents living with perinatally acquired HIV, stratified by stunting at age 10 years, by sex and regions. CIPHER global cohort collaboration, 1994–2015.

Overall, severe immunodeficiency and severe wasting at age 10 was associated with smaller HAZ improvements over time compared to those with no immunodeficiency or wasting. Female adolescents severely stunted at ART initiation had smaller HAZ improvements over time compared to non‐stunted females (Table 2). Across regions, those who initiated ART between age 5 and 10 years had lower HAZ values at age 10 and over time during adolescence compared to those who initiated ART between age 0 and 5 years (Figure S1). Despite significant HAZ improvements over time, particularly in females, those moderately or severely stunted at age 10 did not reach similar values to those non‐stunted at the same age (Figure 3).

**Table 2:** Associated factors to height‐for‐age Z‐score evolution between age 10 and 17 years for males and females: multivariate linear mixed model with fractional polynomials

	For males				For females			
	At 10 years of age		Between 10 and 19 years of age		At 10 years of age		Between 10 and 19 years of age	
Variables	Mean difference (coef (sd))	*p*‐value	Mean difference by year (coef (sd))	*p*‐value	Mean difference (coef (sd))	*p*‐value	Mean difference by year (coef (sd))	*p*‐value
Immunodeficiency at age 10		0.896		**<0.001**		**0.002**		**<0.001**
Moderate versus no	−0.003 (0.015)	0.853	−0.002 (0.001)	0.037	−0.030 (0.017)	0.084	−0.008 (0.004)	0.070
Severe versus no	−0.009 (0.014)	0.502	−0.006 (0.001)	<0.001	−0.047 (0.016)	0.004	−0.012 (0.004)	0.003
Missing versus no	0.002 (0.011)	0.850	−0.001 (0.001)	0.283	0.015 (0.012)	0.212	0.001 (0.004)	0.691
Stunting at ART initiation		**<0.001**		**<0.001**		0.084		**<0.001**
Moderate versus no	−0.032 (0.014)	0.021	0.001 (0.001)	0.191	−0.037 (0.015)	0.013	0.001 (0.004)	0.842
Severe versus no	−0.062 (0.015)	<0.001	0.001 (0.001)	0.485	−0.030 (0.018)	0.094	−0.014 (0.005)	0.003
Missing versus no	−0.021 (0.011)	0.056	0.000 (0.001)	0.891	−0.018 (0.012)	0.140	−0.008 (0.003)	0.012
Wasting at age 10		**<0.001**		**<0.001**		**<0.001**		**<0.001**
Moderate versus no	−0.083 (0.017)	<0.001	−0.007 (0.001)	<0.001	−0.131 (0.020)	<0.001	−0.014 (0.006)	0.012
Severe versus no	−0.160 (0.028)	<0.001	−0.011 (0.002)	<0.001	−0.304 (0.039)	<0.001	−0.052 (0.012)	<0.001
Missing versus no	−0.217 (0.040)	<0.001	−0.025 (0.004)	<0.001	−0.257 (0.046)	<0.001	−0.064 (0.013)	<0.001
Region (vs. North America and Europe)		**0.012**		**<0.001**		**<0.001**		**<0.001**
Central, South America and the Caribbean	−0.034 (0.026)	0.192	0.000 (0.002)	0.995	−0.093 (0.026)	<0.001	0.001 (0.005)	0.833
Asia‐Pacific	−0.042 (0.020)	0.032	0.007 (0.002)	<0.001	−0.057 (0.021)	0.008	−0.003 (0.005)	0.542
West and Central Africa	−0.027 (0.023)	0.232	−0.011 (0.002)	<0.001	−0.039 (0.025)	0.128	−0.013 (0.007)	0.064
Botswana and South Africa	−0.059 (0.017)	<0.001	−0.009 (0.001)	<0.001	−0.098 (0.019)	<0.001	−0.013 (0.005)	0.003
East and Southern Africa (except Botsw. and SA)	−0.054 (0.016)	<0.001	−0.010 (0.001)	<0.001	−0.105 (0.018)	<0.001	−0.035 (0.004)	<0.001

Note: Adjusted on fractional polynomials for time since age 10 (mfp 2,3), age at ART initiation (mfp 2,2) and height‐for‐age Z‐score at age 10 (mfp –1,0). See Figures S1 and S3 for the estimated growth curves by age at ART initiation and stunting at age 10.

Moderate stunting or wasting: HAZ or BAZ = [–3; –2[SD; Severe stunting or wasting: HAZ or BAZ<‐3 SD. Moderate immunodeficiency: CD4 cells/mm^3^ = [250;500[; Severe immunodeficiency: CD4 cells/ mm^3^<250.

CIPHER global cohort collaboration, 1994–2015.

Bold values referred to statistical significance for each variable at a threshold of <0.05.

### 3.3 CD4 evolution during adolescence

**Figure 4:**
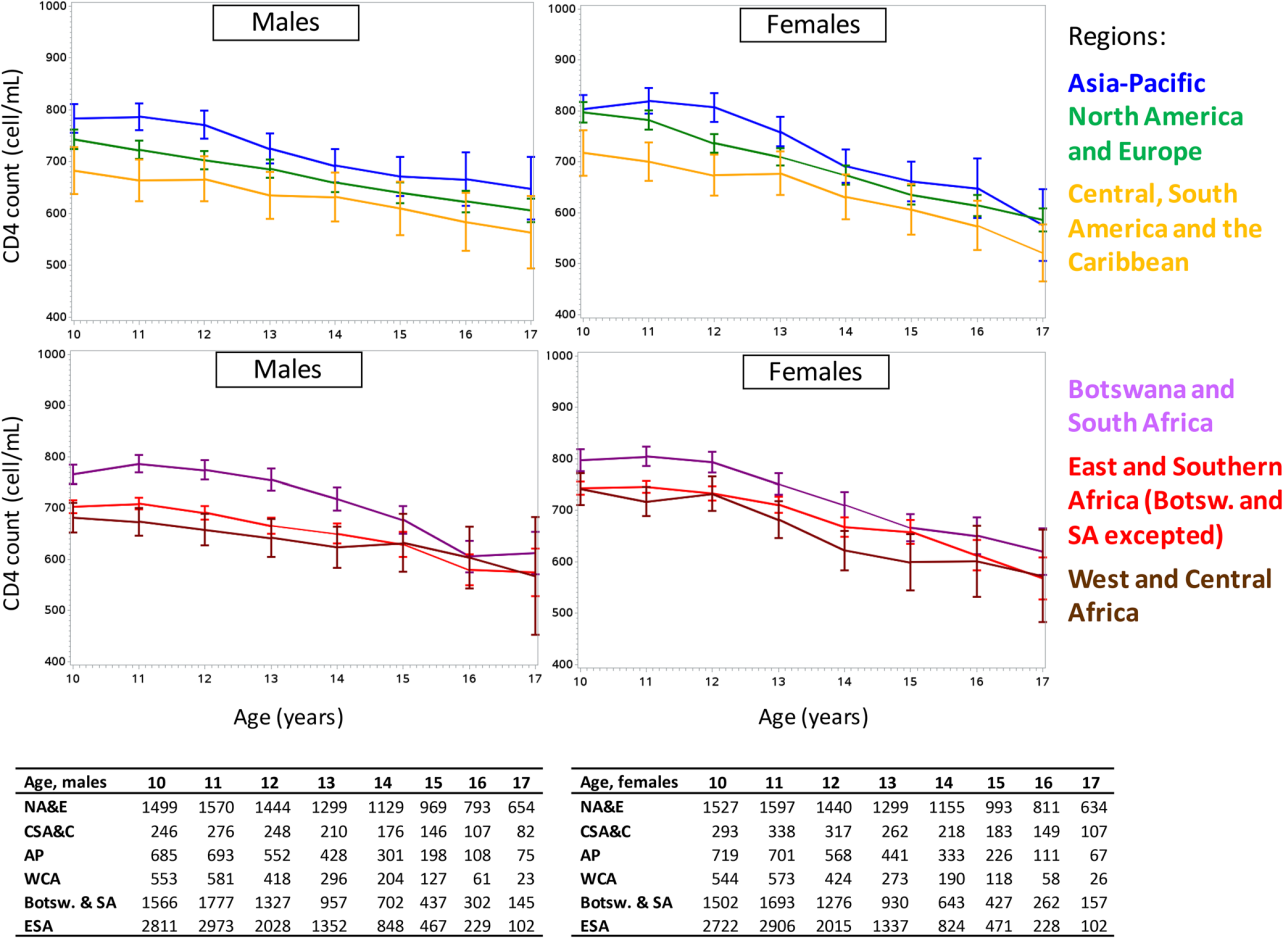
Mean CD4 count evolution between 10 and 19 years of age, stratified by regions and sex, with sample size by year. CIPHER global cohort collaboration, 1994–2015.

All regions followed the same pattern, with a CD4 decline overtime (Figure 4). The same pattern was observed in sensitivity analyses using age‐adjusted CD4 ratio instead of CD4 cells/mm^3^ (Figure S2). From age 10 to age 16 years, mean CD4 counts dropped from 768 to 607 cells/mm^3^ (i.e. 0.78–0.65 in age‐adjusted CD4 ratio).

Adjusted estimates for CD4 counts at age 10 were higher in females, those initiated early on ART and born after year 2000 (Table 3). A CD4 decline was observed whatever the CD4 level at age 10. Those moderately or severely immunodeficient at age 10 had CD4 improvements in the first years of adolescence, followed by a plateau, those non‐immunodeficient at age 10 experienced a CD4 decline, even if overall they remain at higher CD4 count level than the two other groups (Figure S3). APH born before 1996 had lower CD4 counts than those born after, especially with those born after 2000 who had not reached late adolescence by the time the database was closed. Thus, the CD4 decline observed may be partly explained by different data contributions, with higher CD4 count observed before age 15 due to data from those born after 2000, and lower CD4 counts after age 15 because of those born before 2000. However, even adjusting on this parameter, the CD4 decline remained.

**Table 3:** Associated factors to CD4 count evolution (cells/mm^3^), mutlivariate linear mixed model

	At 10 years of age		Between 10 and 19 years of age	
Variables	Mean difference (coef (sd))	*p*‐value	Mean difference by year (coef (sd))	*p*‐value
Sex (girls vs. boys)	41 (5)	**<0.001**	−8 (2)	**<0.001**
Age groups at ART initiation (years)		**<0.001**		**<0.001**
0–2 versus 5–10	79 (8)	<0.001	−7 (3)	<0.001
2–5 versus 5–10	86 (7)	<0.001	−13 (2)	<0.001
Immunodeficiency for age at ART initiation		**<0.001**		**0.005**
Moderate versus no	−99 (9)	<0.001	10 (3)	<0.001
Severe versus no	−126 (7)	<0.001	7 (2)	0.004
Missing versus no	−96 (7)	<0.001	5 (2)	0.034
Immunodeficiency for age at 10 years of age		**<0.001**		**<0.001**
Moderate versus no	−443 (8)	<0.001	53 (3)	<0.001
Severe versus no	−613 (8)	<0.001	82 (2)	<0.001
Missing versus no	−116 (8)	<0.001	4 (3)	0.105
Severity of stunting at 10 years of age		**<0.001**		**<0.001**
Moderately versus no	38 (7)	<0.001	1 (3)	0.765
Severely versus no	19 (11)	0.080	17 (4)	<0.001
Missing versus no	−16 (6)	0.012	9 (2)	<0.001
Year of birth		**<0.001**		**0.011**
<1996 versus [1966–2000[	−41 (8)	<0.001	1 (2)	0.703
≥2000 versus [1996–2000[	25 (6)	<0.001	8 (3)	0.003
Region (vs. North America and Europe)		**<0.001**		0.676
Central, South America and the Caribbean	−28 (13)	0.025	0 (4)	0.996
Asia‐Pacific	18 (11)	0.084	1 (3)	0.816
West and Central Africa	12 (12)	0.299	−5 (4)	0.227
Botswana and South Africa	58 (9)	<0.001	−3 (3)	0.247
East and Southern Africa (except Bostw. and SA)	22 (9)	0.015	−1 (3)	0.651

Note: Moderate stunting or wasting: HAZ or BAZ = [–3; –2[SD; Severe stunting or wasting: HAZ or BAZ<‐3 SD.

Moderate immunodeficiency: CD4 cells/mm^3^ = [250;500[; Severe immunodeficiency: CD4 cells/mm^3^<250.

CIPHER global cohort collaboration, 1994–2015.

Bold values referred to statistical significance for each variable at a threshold of <0.05.

### 3.4 Joint evolution between CD4 and height‐for‐age

Neither of the two statistical methods implemented provided evidence of HAZ and CD4 co‐evolution, even when taking into account the heterogeneity of growth evolution by regions and sex and conducting sub‐group analyses. Correlations between the two outcomes were very low for every sub‐group considered, and no clear patterns of a joint evolution emerged (Supplementary results).

## 4 DISCUSSION

This global analysis conducted in a large sample size representing high‐, middle‐ and low‐income countries allowed an extended description of growth and CD4 patterns of APH enrolled in care between 1994 and 2015. Adolescents living with HIV worldwide experienced different linear growth patterns by region and sex, while their CD4 patterns were similar, showing a decline from the age of 10. Late ART initiation, along with severe stunting and low CD4 cell count at age 10 were significant markers of poor growth and poor immune response during adolescence.

Lower HAZ among children and adolescents living with HIV compared to their HIV‐uninfected peers have been extensively described in both high and low‐and‐middle income countries, with demonstrated benefits of early ART treatment on catch‐up growth [17, 19, 22, 23]. Growth differences observed by region may highlight differences in access to HIV care, with HAZ during adolescence proportionately increasing with the rate of APH receiving ART before the age of 2. The burden of malnutrition in the general population also varies greatly by country‐income groups, which can be an underlying condition increasing the risk of non‐reversible stunting during adolescence. Indeed, repeated exposures to malnutrition during infancy result in growth retardation, and may lead to puberty delay, that can further slowdown height gains [39, 40]. Furthermore, maternal determinants, such as young age and poor nutrition status, are also strong predictors of long‐term stunting in children, and are most commonly found in low‐income groups [40]. In the Asia‐Pacific, the use of WHO growth standards instead of national growth chart references may have resulted in relatively lower HAZ values.

APH experience puberty delays compared to HIV‐uninfected adolescents [41, 42, 43], which may explain the high prevalence of stunting observed in APH. Most studies have been conducted in youth with late access to ART, at similar ages to our participants from sub‐Saharan African regions. Few studies have investigated puberty delays in early treated APH, or explored its association with growth for APH. In a study in the United States, APH had delayed sexual maturity compared to HIV‐exposed but uninfected adolescents, with HAZ mediating the effect of HIV on age at sexual maturity for both males and females [42]. Puberty delays were also observed in APH living in Uganda and Zimbabwe, with greater delays for those stunted [43]. Across regions, growth patterns varied by sex, with more pronounced differences in sub‐Saharan African regions, while males and females had similar characteristics at ART initiation and age 10. We previously highlighted these differences within the IeDEA and the EPPICC networks, from which data are also included in this analysis [17, 23]. In Uganda and Zimbabwe, males had more height disadvantage compared to female APH throughout adolescence [43]. Additional data are needed to further explain these sex differences in growth development for APH, describing the timing of puberty onset, and hormonal and metabolic changes that may be associated with growth.

While growth patterns may be dependent on nutrition and HIV care history during childhood, similar CD4 evolution was observed across settings, with comparable CD4 values at age 10 followed by no CD4 count increase, or even a CD4 decline over time. While this observed decline rarely reached immunodeficiency levels, this result has to be further explored.

First, this decline may be related to low levels of viral suppression or ART adherence across adolescence [24, 28, 44], variables we were not able to measure. Other studies have shown that despite sustained viral suppression, people living with HIV may experience low CD4 count levels and chronic immune activation [45, 46, 47], a potential factor explaining CD4 count depletion [48]. For example, a high proportion of adults living with HIV in sub‐Saharan Africa had suboptimal immune recovery after 6 years of ART in a multicountry prospective cohort [49]; the CD4/CD8 ratio, an indicator of immune activation and inflammation, has been shown to remain low in long‐term ART‐treated adults [50], especially if initiated late on ART [50, 51]. Also, based on this CD4/CD8 ratio, a third of children and adolescents living with HIV who maintained viral load suppression did not achieve immune recovery in a Spanish cohort [52] as well as in a UK cross‐sectional study [53]. Less than half had reached CD4/CD8 ratio normalization in Thailand [54]. A Kenyan study showed high immune activation levels and depleted CD4 percentages [55]. If APH experience suboptimal CD4 counts while being virologically suppressed, that could increase their risk of experiencing opportunistic infections and advanced stage of HIV disease, especially in sub‐Saharan African settings where other infectious diseases are highly prevalent. The phenomenon of chronic immune activation and persistent inflammation is of particular importance as it is associated with higher risk of morbidity and mortality among people living with HIV [56]. The CD4 decline we observed may be due to this phenomenon, but more specific biological measurements are needed to verify this hypothesis.

Another explanation for this CD4 decline would be information bias, with adolescents born before year 2000, mostly from North America and Europe, contributing more CD4 data after 15 years of age compared to those born after 2000. This population may have initiated ART on an older regimen, which was less efficient regarding to long‐term immune recovery. However, even if less pronounced, a CD4 decline was observed while adjusting by year of birth.

Our secondary objective on determining a joint evolution between CD4 and growth did not show any conclusive results, even when stratifying by regions to take into account possible differences in nutritional care that could mitigate the results. Potential height gains due to a recent immune recovery can take time to be observed and then may not be translated easily with joint modelling methods. Future work might investigate how the two outcomes interact by building and testing a causal model. We showed that severe immunodeficiency at age 10 was associated with lower height gains during adolescence. Previous studies on the role of CD4 level in APH final height showed contradictory results, no associations between CD4 percentage and final height were seen in a birth cohort in Thailand [57], while it was positively correlated in a small cohort in the United States [58].

The limitations to this study include that key variables, such as viral load, ART adherence or ART regimen, were not included because of their sporadic documentation across settings. Regarding ART regimen, previous studies did not highlight significant associations between ART regimen at ART initiation and catch‐up growth [40] or final height [57], while its role on long‐term CD4 evolution has not been assessed in APH to our knowledge. A conservative definition of perinatal HIV acquisition was used, with a threshold of 10 years of age at inclusion if not documented. We may have excluded a subset of APH accessing HIV care late, who could have higher rates of stunting and different CD4 profiles due to either slow HIV progression [59] or advanced HIV disease. Children who did not reach adolescence due to loss‐to‐follow‐up or death did not contribute to the data, leading to a survival bias. Data contribution varied across settings between early and late adolescence, median age at last visit was especially low in sub‐Saharan Africa; therefore, the growth and CD4 data observed during late adolescence have to be interpreted with caution as they may come from a selected population. Despite those limitations, this study provides critical evidence on growth and CD4 data for APH, thanks to a large, multi‐regional dataset, developed by a unique research collaboration on paediatric and adolescent HIV.

## 5 CONCLUSIONS

These original results support the importance of closely monitoring growth and CD4 patterns during adolescence, to further develop appropriate and timely interventions allowing APH to reach adulthood as healthy as possible. Our findings on CD4 progression are especially relevant in a time where viral load monitoring is becoming the reference to measure ART effectiveness [60], at the expense of CD4 monitoring that may become less available in low‐and‐middle income countries. Our results advocate for maintaining CD4 count data collection as it provides helpful information on HIV‐related clinical complications [49, 61]. CD4 depletion and the potential role of chronic inflammation on this mechanism [62] is a critical feature to describe and may be a threat for long‐term ART‐treated patients. Even though our results may appear slightly outdated compared to the 2020 recommended paediatric HIV care standards, optimal access to high standard of care remains challenging for some resource‐limited regions that are still facing difficulties to access the most recent and efficient treatment strategies [63]. This multi‐regional, extended description of growth and CD4 patterns, in a large and diverse population of APH, should help further support HIV care for APH worldwide.

## The CIPHER Global Cohort Collaboration

**Project Team**: Julie Jesson* (CERPOP, Inserm, Université de Toulouse, Université Paul Sabatier Toulouse 3, Toulouse, France), Siobhan Crichton* (MRC Clinical Trials Unit, University College London, London, United Kingdom), Matteo Quartagno (MRC Clinical Trials Unit, University College London, London, United Kingdom), Marcel Yotebieng (Division of General Internal Medicine, Department of Medicine, Albert Einstein College of Medicine, Bronx, NY, United States of America), Elaine. J. Abrams (ICAP at Columbia University, Mailman School of Public Health, Columbia University, New York, NY, United States of America), Kulkanya Chokephaibulkit (Siriraj Institute of Clinical Research, Faculty of Medicine, Siriraj Hospital, Mahidol University, Thailand), Sophie Le Coeur (Institut National d'Etude Demographique (INED), Mortality, Health and Epidemiology Unit, Paris, France, Institut de Recherche pour le Developpement (IRD), UMI‐174/PHPT, Faculty of Associated Medical Sciences, Chiang Mai University, Chiang Mai, Thailand), Marie‐Hélène Aké‐Assi (University Hospital Yopougon, Abidjan, Côte d'Ivoire), Kunjal Patel (Harvard T. H. Chan School of Public Health, Boston, Massachusetts, United States of America), Jorge Pinto (School of Medicine, Federal University of Minas Gerais, Belo Horizonte, Brazil), Mary Paul (Baylor International Pediatric AIDS Initiative, Texas Children's Hospital‐USA, Houston, Texas, United States of America), Rachel Vreeman (Department of Global Health, Icahn School of Medicine at Mount Sinai, New York, New York, USA), Mary‐Ann Davies (School of Public Health and Family Medicine, Faculty of Health Sciences, University of Cape Town, Cape Town, South Africa), Ruth Goodall † (MRC Clinical Trials Unit, University College London, London, United Kingdom), Valériane Leroy † (CERPOP, Inserm, Université de Toulouse, Université Paul Sabatier Toulouse 3, Toulouse, France). * Project Statisticians/Epidemiologists; † Project co‐chairs, should be considered joint senior author.

**Data Coordinating Team**: Nicky Maxwell (University of Cape Town, Cape Town, South Africa), Charlotte Duff (MRC Clinical Trials Unit at University College London, London, UK).

**Writing Team**: *CIPHER Steering Committee*: Elaine Abrams (ICAP at Columbia University, Mailman School of Public Health, Columbia University, New York, NY, United States of America), Mary Paul (Baylor International Pediatric AIDS Initiative at Texas Children's Hospital, USA), Jihane Ben‐Farhat (Epicentre, Médecins Sans Frontières, France), Russell Van Dyke (Tulane University Health Sciences Center, USA), Ali Judd (MRC Clinical Trials Unit at UCL, UK), Rachel Vreeman (Department of Global Health, Icahn School of Medicine at Mount Sinai, New York, New York, USA), Lynne Mofenson (Elizabeth Glaser Pediatric AIDS Foundation, USA), Marissa Vicari (International AIDS Society, Switzerland), George Seage III (Harvard T. H. Chan School of Public Health, USA), Linda‐Gail Bekker (Desmond Tutu HIV Centre, University of Cape Town, South Africa), Shaffiq Essajee (UNICEF), Diana Gibb (MRC Clinical Trials Unit at University College London, London, UK);

*CIPHER Executive Committee*: Lynne Mofenson (Elizabeth Glaser Pediatric AIDS Foundation, USA), Linda‐Gail Bekker (Desmond Tutu HIV Centre, University of Cape Town, South Africa), Marissa Vicari (International AIDS Society, Switzerland), Shaffiq Essajee (UNICEF), Martina Penazzato (World Health Organization, Switzerland);

*CIPHER Project Oversight Group*: Intira Jeannie Collins (MRC Clinical Trials Unit at UCL, UK), Kara Wools‐Kaloustian (Indiana University School of Medicine, USA), Mary‐Ann Davies (University of Cape Town, South Africa), Valériane Leroy (CERPOP, Inserm, Université de Toulouse, Université Paul Sabatier Toulouse 3, Toulouse, France), Ruth Goodall (MRC Clinical Trials Unit at UCL, UK), Kunjal Patel (Harvard T. H. Chan School of Public Health, USA), Ali Judd (MRC Clinical Trials Unit at UCL, UK), Rachel Vreeman (Department of Global Health, Icahn School of Medicine at Mount Sinai, New York, New York, USA), Amy Slogrove (Department of Paediatrics & Child Health, Faculty of Medicine & Health Sciences, Stellenbosch University, Worcester, South Africa), Kate Powis (Harvard T. H. Chan School of Public Health, USA), Paige Williams (Harvard T. H. Chan School of Public Health, USA), Siobhan Crichton (MRC Clinical Trials Unit at UCL, UK), Julie Jesson (CERPOP, Inserm, Université de Toulouse, Université Paul Sabatier Toulouse 3, Toulouse, France), George Seage III (Harvard T. H. Chan School of Public Health, USA);

*BIPAI*: Mogomotsi Matshaba (Baylor College of Medicine Children's Foundation‐Botswana), Lineo Thahane (Baylor College of Medicine Children's Foundation‐Lesotho), Phoebe Nyasulu (Baylor College of Medicine Children's Foundation‐Malawi), Bhekumusa Lukhele (Baylor College of Medicine Children's Foundation‐eSwatini), Lumumba Mwita (Baylor College of Medicine Children's Foundation‐Tanzania), Adeodata Kekitiinwa‐Rukyalekere (Baylor College of Medicine Children's Foundation – Uganda), Sebastian Wanless (Baylor International Pediatric AIDS Initiative at Texas Children's Hospital, Data Manager);

*EPPICC*: Siobhan Crichton (MRC Clinical Trials Unit at University College London, London, UK), Matteo Quartagno (MRC Clinical Trials Unit at University College London, London, UK), Intira Jeannie Collins (MRC Clinical Trials Unit at University College London, London, UK), Ruth Goodall (MRC Clinical Trials Unit at University College London, London, UK), Tessa Goetghebuer (Hospital St Pierre, Brussels, Belgium), Claire Thorne (UCL Great Ormond Street Institute of Child Health, University College London, UK), Josiane Warszawski (Inserm U1018, Centre de recherche en Epidémiologie et Santé des Populations, France), Luisa Galli (Infection Disease Unit, Meyer Children's University Hospital, Florence, Italy, Department of Health Sciences, University of Florence, Italy), Annemarie van Rossum (Erasmus MC University Medical Center Rotterdam‐Sophia Children's Hospital, Rotterdam, The Netherlands), Diana M Gibb (MRC Clinical Trials Unit at University College London, London, UK), Carlo Giaquinto (Padova University/ PENTA Foundation, Italy), Magdalena Marczynska (Medical University of Warsaw, Hospital of Infectious Diseases in Warsaw, Poland), Laura Marques (Centro Hospitalar do Porto, Portugal), Filipa Prata (Hospital de Santa Maria, Lisboa, Portugal), Luminita Ene (Victor Babes Hospital, Bucharest, Romania), Lyuba Okhonskaya (Republican Hospital of Infectious Diseases, St Petersburg, Russian Federation), Marisa Navarro (Hospital General Universitario “Gregorio Marañón”, Madrid, Spain), Antoinette Frick (Hospital Universitari Vall d' Hebron, Vall d' Hebron Research Institute, Universitat Autònoma de Barcelona; Barcelona, Spain), Lars Naver (Karolinska University Hospital and Karolinska Institutet, Stockholm, Sweden), Christian Kahlert (Children's Hospital of Eastern Switzerland, Saint Gallen, Switzerland), Sophie Le Coeur (Institut National d'Etude Demographique (INED), Mortality, Health and Epidemiology Unit, Paris, France, Institut de Recherche pour le Developpement (IRD), UMI‐174/PHPT, Faculty of Associated Medical Sciences, Chiang Mai University, Chiang Mai, Thailand), Ali Judd (MRC Clinical Trials Unit at University College London, London, UK), Alla Volokha (Shupyk National Medical Academy of Postgraduate Education, Kiev), Elizabeth Chappell (MRC Clinical Trials Unit at University College London, London, UK);

*IeDEA‐CCASAnet*: Jorge Pinto (Department of Pediatrics, School of Medicine, Federal University of Minas Gerais, Brazil) Jean William Pape (GHESKIO Center, Port‐au‐Prince, Haiti), Vanessa Rouzier (GHESKIO Center, Port‐au‐Prince, Haiti), Adias Marcelin (GHESKIO Center, Port‐au‐Prince, Haiti), Regina Succi (Universidade Federal de Sao Paulo, Brazil);

*IeDEA Asia‐Pacific*: Kulkanya Chokephaibulkit (Faculty of Medicine, Siriraj Hospital, Mahidol University, Bangkok, Thailand), Annette H Sohn (TREAT Asia/amfAR, Bangkok, Thailand), Azar Kariminia (Kirby Institute, University of New South Wales, Sydney, Australia);

*IeDEA Central Africa*: Marcel Yotebieng (Division of General Internal Medicine, Department of Medicine, Albert Einstein College of Medicine, Bronx, NY, United States of America), Andrew Edmonds (Gillings School of Global Public Health, University of North Carolina at Chapel Hill, USA), Patricia Lelo (Pediatric Hospital Kalembe Lembe, Lingwala, Kinshasa, Demogratic Republic of Congo);

*IeDEA East Africa*: Rita Lyamuya (Morogoro Regional Hospital, Morogoro, Tanzania), Edith Apondi Ogalo (Moi Teaching and Referral Hospital, Kenya), Francesca Akoth Odhiambo (Center for Microbiology Research, Kenya Medical Research Institute, Nairobi, Kenya), Kara Wools‐Kaloustian (Indiana University School of Medicine, Department of Medicine, Division of Infectious Diseases, Indianapolis, Indiana), Rachel Vreeman (Department of Global Health, Icahn School of Medicine at Mount Sinai, New York, New York, USA);

*IeDEA Southern Africa*: Andreas D Haas (Institute of Social and Preventive Medicine, University of Bern, Switzerland), Carolyn Bolton (Centre for Infectious Disease Research in Zambia, Lusaka, Zambia), Josephine Muhairwe (SolidarMed, Lesotho, Mozambique, Zimbabwe), Hannock Tweya (Lighthouse Trust Clinic, Lilongwe, Malawi), Mary‐Ann Davies (Center for Infectious Diseases Epidemiology and Research, University of Cape Town, Cape Town, South Africa);

*IeDEA West Africa*: Mariam Sylla (CHU Gabriel Toure, Bamako, Mali), Marceline D'Almeida, (Centre National Hospitalier Universitaire Hubert K. Maga, Cotonou, Bénin), Lorna Renner (Korle Bu Teaching Hospital, Accra, Ghana), Marie‐Hélène Aké‐Assi (University Hospital Yopougon, Abidjan, Côte d'Ivoire), Valeriane Leroy (CERPOP, Inserm, Université de Toulouse, Université Paul Sabatier Toulouse 3, Toulouse, France);

*IMPAACT/PHACS*: Mark J Abzug (University of Colorado School of Medicine and Children's Hospital Colorado, USA), Paige Williams (Harvard T. H. Chan School of Public Health, USA), James Oleske (Rutgers ‐ New Jersey Medical School, USA), Murli Purswani (Bronx‐Lebanon Hospital Center, USA), George Seage III (Harvard T. H. Chan School of Public Health, USA), Russell Van Dyke (Tulane University, USA), Kunjal Patel (Harvard T. H. Chan School of Public Health, USA);

*ICAP‐Optimal Models*: Elaine Abrams (ICAP‐Columbia University, Mailman School of Public Health, USA), Chloe Teasdale (ICAP‐Columbia University, Mailman School of Public Health, USA), Harriet Nuwagaba‐Biribonwoha (ICAP‐Columbia University, Mailman School of Public Health, USA).

We would like to acknowledge George R. Seage III (1957–2021) for all of his contributions to the CIPHER Global Cohort Collaboration as the Principal Investigator of the Harvard CIPHER data center and the Pediatric HIV/AIDS Cohort Study Data and Operations Center. His leadership, collaborative spirit and contributions to understanding the long‐term effects of perinatal HIV infection and antiretroviral treatment have improved care and antiretroviral drug safety guidelines for children, young adults and families all over the world. He is sorely missed by the CIPHER community. The full list of acknowledgements is available as supporting information (Data S1).

## COMPETING INTERESTS

MV's work at CIPHER is funded through Unrestricted Educational grants received from ViiV Healthcare and Janssen to the International AIDS Society. AS's institution receives research and travel funding from ViiV Healthcare. CT has received grant funding from ViiV Healthcare via the Penta Foundation (to UCL), and personally from ViiV for participation on an advisory board. AJ reports grants from Abbvie, Bristol Myers Squibb, Gilead, Janssen Pharmaceuticals and ViiV Healthcare through the PENTA Foundation, and from the European and Developing Countries Clinical Trials Partnership, Gilead Sciences, the International AIDS Society, NHS England, Medical Research Council and PENTA Foundation outside the submitted work. All monies were paid to her institution. IJC received grants from the following companies (via her institution) in the past 3 years: ViiV, AbbVie and Gilead.

All remaining authors declare no competing interests.

## AUTHORS’ CONTRIBUTIONS

JJ conducted the analysis and wrote the paper, with methodological support from SC and MQ and under the supervision of VL and RG. JJ, SC and RG contributed to the study design and protocol development. MY represents the IeDEA Central African cohort. EA and KP represent the IMPAACT and PHACS cohort and EA represents Optimal Models. KC represents the IeDEA Asia‐Pacific cohort. MhAa, VL and JJ represent the IeDEA West African cohort. JP represents CCASAnet. SLC, RG and SC represent the EPPICC cohort. MP represents BIPAI. RV represents the IeDEA East Africa cohort. MaD represents the IeDEA Southern Africa cohort.

All co‐authors participated to the interpretation of the results, and subsequently revised the manuscript. All authors listed as belonging to the Project Team, Data Coordinating Team and the Writing Team have contributed sufficiently to the conception, design, data collection, analysis, writing and/or review of the manuscript to take public responsibility for it. All authors have read and approved the final manuscript.

## FUNDING

This work was supported by the International AIDS Society – Collaborative Initiative for Paediatric HIV Education & Research (IAS‐CIPHER, http://www.iasociety.org/CIPHER), which is made possible through funding from CIPHER Founding Sponsor ViiV Healthcare (https://www.viivhealthcare.com) and Janssen (http://www.janssen.com).

Individual networks contributing to the CIPHER Cohort Collaboration have received the following financial support: CCASAnet receives funding from the United States (US) National Institutes of Health (NIH) (grant number U01AI069923); IeDEA Asia Pacific receives funding from the US NIH (grant number U01AI069907); IeDEA Central Africa receives funding from the US NIH (grant number U01AI096299); IeDEA East Africa receives funding from the US NIH (grant number U01A1069911); IeDEA Southern Africa receives funding from the US NIH (grant number U01AI069924); IeDEA West Africa receives funding from the US NIH (grant number U01AI069919); overall support for the International Maternal Pediatric Adolescent AIDS Clinical Trials Network (IMPAACT) was provided by the National Institute of Allergy and Infectious Diseases (NIAID) with co‐funding from the Eunice Kennedy Shriver National Institute of Child Health and Human Development (NICHD) and the National Institute of Mental Health (NIMH), all components of the NIH, under Award Numbers UM1AI068632 (IMPAACT LOC), UM1AI068616 (IMPAACT SDMC) and UM1AI106716 (IMPAACT LC), and by NICHD contract number HHSN275201800001I; PHACS receives funding from the US NIH (grant numbers U01 HD052102 and U01 HD052104); the Optimal Models (ICAP at Columbia University) project was supported by the President's Emergency Plan for AIDS Relief (PEPFAR) through the Centers for Disease Control and Prevention (cooperative agreement numbers: 5U62PS223540 and 5U2GPS001537); EPPICC receives funding from the PENTA Foundation (http://penta‐id.org), and received support from the European Union's Horizon 2020 research and innovation programme under grant agreement no 825579 for the REACH study. The MRC Clinical Trials Unit at UCL is supported by the Medical Research Council (programme number MC_UU_12023/26). Individual cohorts contributing to EPPICC receive the following support: the ATHENA database is maintained by Stichting HIV Monitoring and supported by a grant from the Dutch Ministry of Health, Welfare and Sport through the Centre for Infectious Disease Control of the National Institute for Public Health and the Environment. CoRISPE‐cat receives financial support from the Instituto de Salud Carlos III through the Red Temática de Investigación Cooperativa en Sida (grant numbers RED RIS RD06/0006/0035 yRD06/0006/0021). Financial support for CoRISpeS and Madrid Cohort was provided by the Instituto de Salud Carlos III through the Red Tematica de Investigacion Cooperativa en Sida (RED‐RIS) project (grant number RD16/0025/0019) as part of the Plan R+D+I and cofinanced by ISCIII‐Subdireccion General de Evaluación and Fondo Europeo de Desarrollo Regional (FEDER). FIS PI19/01530. The Swiss HIV Cohort Study is supported by the Swiss National Science Foundation (grant number: 177499) and by the SHCS Research Foundation. The Thai cohort study was funded by the Global Fund to fight AIDS, Tuberculosis and Malaria, Thailand (PR‐A‐N‐008); Oxfam Great Britain, Thailand (THAA51); Ministry of Public Health, Thailand; and Institut de Recherche pour le Développement (IRD), France. Ukraine Paediatric HV Cohort is supported by the PENTA Foundation. CHIPS is funded by the NHS (London Specialized Commissioning Group) and has received additional support from Bristol‐Myers Squibb, Boehringer Ingelheim, GlaxoSmithKline, Roche, Abbott and Gilead Sciences. The MRC Clinical Trials Unit at UCL is supported by the Medical Research Council (programme number MC_UU_12023/26).

No funding bodies had any role in study design, data collection and analysis, decision to publish or preparation of the manuscript.

## DISCLAIMER

This work is solely the responsibility of the authors and does not necessarily represent the official views of any of the institutions mentioned above.

## Supporting information


**Additional files**: Additional information may be found under the Supporting Information tab for this article.
**Table S1**: Characteristics of study participants for the growth and the CD4 analyses, by regions.
**Table S2**: Characteristics of study participants for the growth and the CD4 analyses, by regions.
**Figure S1**: Adjusted estimated mean height‐for‐age Z‐scores for adolescents living with perinatally acquired HIV, stratified by age at ART initiation, according to sex and regions.
**Figure S2**: Mean CD4 evolution during adolescence for adolescents living with perinatally acquired HIV, stratified by sex, expressed in cells/mm^3^ (left) and in age‐adjusted ratio (right).
**Figure S3**: Mean CD4 evolution during adolescence for adolescents living with perinatally acquired HIV, stratified by immunodeficiency at ART initiation and at age 10, as well as by age at ART initiation and year of birth.
**Supplementary results**: Joint modelling of growth and immunology, illustrations for males, for two regions (North America and Europe, East and Southern Africa excluding Botswana and South Africa)

**Data S1**: Full list of acknowledgments for the CIPHER Global Cohort Collaboration

## Data Availability

Data are accessible in principle by applying to the Collaborative Initiative for Paediatric HIV Education and Research (CIPHER) Global Cohort Collaboration Data Centres. The CIPHER Global Cohort Collaboration is a multinetwork, multisite collaboration and this study combined data from different sites. The data do not belong to the CIPHER Global Cohort Collaboration itself; data ownership remains with the participating sites. Each site has approval from its own local Institutional Review Board to collect routine data on patients and to transfer those data anonymously to the CIPHER Global Cohort Collaboration Project University of Cape Town Research Centre (Cape Town, South Africa). For some sites and networks, Institutional Review Board approval for use of this data is restricted to the specific protocols approved to protect patient identities. Requests for access to data can be directed to the corresponding author.
