# Supplementary material for: Growth and CD4 patterns of adolescents living with perinatally acquired HIV worldwide, a CIPHER cohort collaboration analysis
Source: J Int AIDS Soc. 2022 Mar 7;25(3):e25871. doi: 10.1002/jia2.25871 (PMC8901148; doi:10.1002/jia2.25871)
Supplement: Supplementary file 1 — Additional files: Additional information may be found under the Supporting Information tab for this article. Table S1: Characteristics of study participants for the growth and the CD4 analyses, by regions. Table S2: Characteristics of study participants for the growth and the CD4 analyses, by regions. Figure S1: Adjusted estimated mean height‐for‐age Z‐scores for adolescents living with perinatally acquired HIV, stratified by age at ART initiation, according to sex and regions. Figure S2: Mean CD4 evolution during adolescence for adolescents living with perinatally acquired HIV, stratified by sex, expressed in cells/mm3 (left) and in age‐adjusted ratio (right). Figure S3: Mean CD4 evolution during adolescence for adolescents living with perinatally acquired HIV, stratified by immunodeficiency at ART initiation and at age 10, as well as by age at ART initiation and year of birth. Supplementary results: Joint modelling of growth and immunology, illustrations for males, for two regions (North America and Europe, East and Southern Africa excluding Botswana and South Africa) [file JIA2-25-e25871-s002.docx]

| **Supplementary Table 1: Characteristics of study participants for the Growth and the CD4 analyses, by regions. CIPHER global cohort collaboration, 1994-2015** | | | | | | | | | | | | | | | | | | | | | |
| --- | --- | --- | --- | --- | --- | --- | --- | --- | --- | --- | --- | --- | --- | --- | --- | --- | --- | --- | --- | --- | --- |
| **Characteristics**  **Growth analyses** | **North America and Europe**  **(N=3352)** | | | **Central,**  **South America**  **and the Caribbean**  **(N=719)** | | **Asia –**  **Pacific**  **(N=1551)** | | | **West and**  **Central Africa**  **(N=1394)** | | | **East and Southern Africa (except Botsw and SA)**  **(N=11,181)** | | | | **Botswana**  **and South Africa**  **(N=2742)** | | | | **p-value*** | |
| **At ART initiation** |  |  | |  |  |  | |  |  |  | | |  | |  | |  | |  | |  |
| **Sex, n %** |  |  | |  |  |  | |  |  |  | | |  | |  | |  | |  | | 0.162 |
| Female | 1693 | 51 | | 388 | 54 | 783 | | 50 | 692 | 50 | | | 5539 | | 50 | | 1332 | | 49 | |  |
| Male | 1659 | 49 | | 331 | 46 | 768 | | 50 | 702 | 50 | | | 5642 | | 50 | | 1410 | | 51 | |  |
| **Age, median (IQR)** | 2.4 (0.6 ; 5.7) | | | 4.3 (2.0 ; 6.7) | | 5.5 (3.4 ; 7.4) | | | 7.5 (5.7 ; 8.8) | | | 7.6 (6.0 ; 8.9) | | | | 6.9 (4.6 ; 8.5) | | | | <0.001 | |
| **Age groups (years), n %** |  |  | |  |  |  | |  |  |  | | |  | |  | |  | |  | | <0.001 |
| 0-2 | 1551 | 46 | | 180 | 25 | 220 | | 14 | 17 | 1 | | | 124 | | 1 | | 299 | | 11 | |  |
| 2-5 | 815 | 24 | | 235 | 33 | 443 | | 29 | 229 | 16 | | | 1590 | | 14 | | 483 | | 18 | |  |
| 5-10 | 986 | 29 | | 304 | 42 | 888 | | 57 | 1148 | 82 | | | 9467 | | 85 | | 1960 | | 71 | |  |
| **HAZ, median (IQR)** | -0.95  (-1.81 ; -0.06) | | | -1.74  (-2.72 ; -0.76) | | -2.38  (-3.33 ; -1.48) | | | -1.77  (-2.72 ; -0.76) | | | -2.05  (-2.98 ; -1.17) | | | | -2.05  (-2.85 ; -1.19) | | | | <0.001 | |
| **Severity of stunting, n %** |  |  | |  |  |  | |  |  |  | | |  | |  | |  | |  | | <0.001 |
| None | 981 | 29 | | 220 | 31 | 399 | | 26 | 443 | 32 | | | 2817 | | 25 | | 925 | | 34 | |  |
| Moderate | 167 | 5 | | 103 | 14 | 288 | | 19 | 185 | 13 | | | 1544 | | 14 | | 569 | | 21 | |  |
| Severe | 92 | 3 | | 68 | 9 | 333 | | 21 | 143 | 10 | | | 1422 | | 13 | | 420 | | 15 | |  |
| *Missing* | 2112 | 63 | | 328 | 46 | 531 | | 34 | 623 | 45 | | | 5398 | | 48 | | 828 | | 30 | |  |
| **WHZ/BAZ, median (IQR)** | 0.27  (-0.52 ; 1.02) | | | -0.36  (-1.24 ; 0.41) | | -0.83  (-1.79 ; -0.07) | | | -1.09  (-2.03 ; -0.23) | | | -0.58  (-1.41 ; 0.14) | | | | -0.46  (-1.21 ; 0.33) | | | | <0.001 | |
| **Severity of wasting, n %** |  |  | |  |  |  | |  |  |  | | |  | |  | |  | |  | | <0.001 |
| None | 1174 | 35 | | 343 | 48 | 801 | | 52 | 566 | 41 | | | 4923 | | 44 | | 1685 | | 61 | |  |
| Moderate | 31 | 1 | | 29 | 4 | 126 | | 8 | 114 | 8 | | | 525 | | 5 | | 134 | | 5 | |  |
| Severe | 21 | 1 | | 17 | 2 | 92 | | 6 | 80 | 6 | | | 272 | | 2 | | 82 | | 3 | |  |
| *Missing* | 2126 | 63 | | 330 | 46 | 532 | | 34 | 634 | 45 | | | 5431 | | 49 | | 841 | | 31 | |  |
| **Immunodeficiency level by age**†**, n %** |  |  | |  |  |  | |  |  |  | | |  | |  | |  | |  | |  |
| None | 924 | 28 | | 110 | 15 | 166 | | 11 | 222 | 16 | | | 1189 | | 11 | | 329 | | 12 | |  |
| Moderate | 531 | 16 | | 101 | 14 | 202 | | 13 | 166 | 12 | | | 780 | | 7 | | 310 | | 11 | |  |
| Severe | 665 | 20 | | 196 | 27 | 782 | | 50 | 530 | 38 | | | 2834 | | 25 | | 1004 | | 37 | |  |
| *Missing* | 1232 | 37 | | 312 | 43 | 401 | | 26 | 476 | 34 | | | 6378 | | 57 | | 1099 | | 40 | |  |
| **At 10 years of age** |  |  | |  |  |  | |  |  |  | | |  | |  | |  | |  | |  |
| **HAZ, median (IQR)** | -0.35  (-1.13 ; 0.41) | | | -1.10  (-1.89 ;-0.34) | | -1.78  (-2.52 ; -1.06) | | | -1.36  (-2.16 ; -0.61) | | | -1.73  (-2.50 ; -0.98) | | | | -1.54  (2.30 ; -0.89) | | | | <0.001 | |
| **Severity of stunting, n %** |  |  | |  |  |  | |  |  |  | | |  | |  | |  | |  | |  |
| No | 2308 | 69 | | 430 | 60 | 809 | | 52 | 713 | 51 | | | 5390 | | 48 | | 1649 | | 60 | |  |
| Moderate | 161 | 5 | | 94 | 13 | 415 | | 27 | 229 | 16 | | | 2544 | | 23 | | 618 | | 22 | |  |
| Severe | 42 | 1 | | 27 | 4 | 187 | | 12 | 72 | 5 | | | 1205 | | 11 | | 222 | | 8 | |  |
| *Missing* | 841 | 25 | | 168 | 23 | 140 | | 9 | 380 | 27 | | | 2042 | | 18 | | 253 | | 9 | |  |
| **BAZ, median (IQR)** | 0.26  (-0.47 ; 1.06) | | | -0.27  (-0.91 ; 0.35) | | -0.83  (-1.48 ; -0.16) | | | -0.86  (-1.56 ; -0.22) | | | -0.63  (-1.31 ; -0.01) | | | | -0.41  (-1.07 ; 0.27) | | | | <0.001 | |
| **Severity of wasting, n %** |  |  | |  |  |  | |  |  |  | | |  | |  | |  | |  | | <0.001 |
| None | 2459 | 73 | | 527 | 73 | 1236 | | 80 | 818 | 59 | | | 8087 | | 72 | | 2328 | | 85 | |  |
| Moderate | 35 | 1 | | 14 | 2 | 131 | | 8 | 115 | 8 | | | 673 | | 6 | | 124 | | 5 | |  |
| Severe | 7 | 0 | | 4 | 1 | 36 | | 2 | 37 | 3 | | | 225 | | 2 | | 32 | | 1 | |  |
| *Missing* | 851 | 25 | | 174 | 24 | 148 | | 10 | 424 | 30 | | | 2196 | | 20 | | 258 | | 9 | |  |
| **CD4 count, median (IQR)** | 753  (529 ; 1001) | | | 685  (438 ; 982) | | 779  (564 ; 1015) | | | 687  (441 ; 973) | | | 700  (458 ; 994) | | | | 729  (451 ; 1036) | | | | <0.001 | |
| **Immunodeficiency level, n %** |  |  | |  |  |  | |  |  |  | | |  | |  | |  | |  | | <0.001 |
| None | 1863 | 56 | | 326 | 45 | 1071 | | 69 | 589 | 42 | | | 776 | | 7 | | 1519 | | 55 | |  |
| Moderate | 276 | 8 | | 72 | 10 | 137 | | 9 | 111 | 8 | | | 667 | | 6 | | 229 | | 8 | |  |
| Severe | 266 | 8 | | 80 | 11 | 140 | | 9 | 150 | 11 | | | 3551 | | 32 | | 405 | | 15 | |  |
| *Missing* | 947 | 28 | | 241 | 34 | 203 | | 13 | 544 | 39 | | | 6187 | | 55 | | 589 | | 21 | |  |
| **Follow-up and cohort profile** |  |  | |  |  |  | |  |  |  | | |  | |  | |  | |  | |  |
| **Year of birth, n %** |  | | |  | |  | | |  | | |  | | | |  | | | | <0.001 | |
| <1996 | 1901 | | 57 | 183 | 25 | 131 | 8 | | 86 | | 6 | 135 | | 1 | | 186 | | 7 | |  | |
| [1996-2000[ | 827 | | 25 | 297 | 41 | 531 | 34 | | 635 | | 45 | 3453 | | 31 | | 1174 | | 43 | |  | |
| >2000 | 624 | | 19 | 239 | 33 | 889 | 57 | | 673 | | 48 | 7593 | | 68 | | 1382 | | 50 | |  | |
| **Age at last visit, median (IQR)** | 15.2  (12.9 ; 17.6) | | | 14.9  (12.8 ; 17.4) | | 13.2  (11.5 ; 14.9) | | | 12.4  (11.1 ; 14.1) | | | 12.3  (11.1 ; 14.0) | | | | 12.9  (11.4 ; 15.0) | | | | <0.001 | |
| **Status at last visit** |  |  | |  |  |  | |  |  |  | | |  | |  | |  | |  | |  |
| Alive, in follow-up | 1677 | 50 | | 537 | 75 | 1281 | | 83 | 1202 | 86 | | | 7398 | | 66 | | 1708 | | 62 | | <0.001 |
| Lost-to-Follow-up‡ | 1046 | 31 | | 93 | 13 | 160 | | 10 | 124 | 9 | | | 2110 | | 19 | | 250 | | 9 | |  |
| Transferred | 587 | 18 | | 63 | 9 | 81 | | 5 | 34 | 2 | | | 1491 | | 13 | | 766 | | 28 | |  |
| Dead | 42 | 1 | | 26 | 4 | 29 | | 2 | 34 | 2 | | | 182 | | 2 | | 18 | | 1 | |  |
| **Patient data contribution, n obs %** |  |  | |  |  |  | |  |  |  | | |  | |  | |  | |  | | <0.001 |
| Between 10 and 14 years | 36,865 | 12 | | 13,334 | 4 | 23,338 | | 7 | 15,525 | 5 | | | 188,656 | | 60 | | 38,099 | | 12 | |  |
| Between 15 and 17 years | 7125 | 26 | | 2748 | 10 | 1909 | | 7 | 1073 | 4 | | | 9547 | | 34 | | 5358 | | 20 | |  |
| ART: Antiretroviral therapy, BAZ: BMI-for-age Z-score, IQR: Interquartile Range, HAZ: Height-for-age Z-score, WHZ: Weight-for-Height Z-score.  *Chi-square or Kruskal-Wallis tests. † WHO 2006 guidelines. ‡ Defined as no contact for more than 365 days or documented loss-to-follow-up. | | | | | | | | | | | | | | | | | | | | | |
| **Supplementary Table 2: Characteristics of study participants for the Growth and the CD4 analyses, by regions. CIPHER global cohort collaboration, 1994-2015** | | | | | | | | | | | | | | | | | | | | | |
| **Characteristics**  **CD4 analyses** | **North America and Europe**  **(N=3567)** | | | **Central,**  **South America**  **and the Caribbean**  **(N=701)** | | **Asia –**  **Pacific**  **(N=1586)** | | | **West and**  **Central Africa**  **(N=1451)** | | | **East and Southern Africa (except Botsw and SA)**  **(N=7605)** | | | | **Botswana**  **and South Africa**  **(N=4647)** | | | | **p-value*** | |
| **At ART initiation** |  |  | |  |  |  | |  |  |  | | |  | |  | |  | |  | |  |
| **Sex, n %** |  |  | |  |  |  | |  |  |  | | |  | |  | |  | |  | | 0.058 |
| Female | 1807 | 51 | | 382 | 54 | 794 | | 50 | 717 | 49 | | | 3742 | | 49 | | 2265 | | 49 | |  |
| Male | 1755 | 49 | | 319 | 46 | 792 | | 50 | 734 | 51 | | | 3863 | | 51 | | 2387 | | 51 | |  |
| **Age, median (IQR)** | 2.5 (0.6 ; 5.8) | | | 4.4 (2.1 ; 6.8) | | 5.6 (3.5 ; 7.4) | | | 7.5 (5.7 ; 8.8) | | | 7.7 (6.2 ; 8.9) | | | | 7.1 (5.1 ; 8.6) | | | | <0.001 | |
| **Age groups (years), n %** |  |  | |  |  |  | |  |  |  | | |  | |  | |  | |  | | <0.001 |
| 0-2 | 1622 | 45 | | 166 | 24 | 214 | | 13 | 15 | 1 | | | 53 | | 1 | | 309 | | 7 | |  |
| 2-5 | 869 | 24 | | 227 | 32 | 447 | | 28 | 240 | 17 | | | 922 | | 12 | | 772 | | 17 | |  |
| 5-10 | 1076 | 30 | | 308 | 44 | 925 | | 58 | 1196 | 82 | | | 6630 | | 87 | | 3566 | | 77 | |  |
| **HAZ, median (IQR)** | -0.95  (-1.81 ; -0.06) | | | -1.75  (-2.74 ; -0.81) | | -2.38  (-3.32 ; -1.48) | | | -1.76  (-2.74 ; -0.91) | | | -2.06  (-2.96 ; -1.19) | | | | -2.04  (-2.86 ; -1.19) | | | | <0.001 | |
| **Severity of stunting, n %** |  |  | |  |  |  | |  |  |  | | |  | |  | |  | |  | | <0.001 |
| None | 976 | 27 | | 212 | 30 | 385 | | 24 | 397 | 27 | | | 1920 | | 25 | | 865 | | 19 | |  |
| Moderate | 164 | 5 | | 101 | 14 | 278 | | 18 | 167 | 12 | | | 1109 | | 15 | | 519 | | 11 | |  |
| Severe | 91 | 3 | | 68 | 10 | 319 | | 20 | 126 | 9 | | | 955 | | 13 | | 388 | | 8 | |  |
| *Missing* | 2336 | 65 | | 320 | 46 | 604 | | 38 | 761 | 52 | | | 3621 | | 48 | | 2875 | | 62 | |  |
| **WHZ/BAZ, median (IQR)** | 0.28  (-0.52 ; 1.02) | | | -0.39  (-1.24 ; 0.40) | | -0.84  (-1.80 ; -0.08) | | | -1.12  (-2.07 ; -0.27) | | | -0.60  (-1.42 ; 0.09) | | | | -0.46  (-1.21 ; 0.33) | | | | <0.001 | |
| **Severity of wasting, n %** |  |  | |  |  |  | |  |  |  | | |  | |  | |  | |  | | <0.001 |
| None | 1168 | 33 | | 335 | 48 | 769 | | 48 | 496 | 34 | | | 3386 | | 45 | | 1563 | | 34 | |  |
| Moderate | 31 | 1 | | 28 | 4 | 123 | | 8 | 105 | 7 | | | 365 | | 5 | | 123 | | 3 | |  |
| Severe | 19 | 1 | | 16 | 2 | 87 | | 5 | 75 | 5 | | | 190 | | 2 | | 75 | | 2 | |  |
| *Missing* | 2349 | 66 | | 322 | 46 | 607 | | 38 | 775 | 53 | | | 3664 | | 48 | | 2886 | | 62 | |  |
| **Immunodeficiency level by age**†**, n %** |  |  | |  |  |  | |  |  |  | | |  | |  | |  | |  | |  |
| None | 951 | 27 | | 121 | 17 | 166 | | 10 | 245 | 17 | | | 1253 | | 16 | | 357 | | 8 | |  |
| Moderate | 544 | 15 | | 116 | 17 | 200 | | 13 | 187 | 13 | | | 838 | | 11 | | 324 | | 7 | |  |
| Severe | 684 | 19 | | 222 | 32 | 783 | | 49 | 564 | 39 | | | 3076 | | 40 | | 1066 | | 23 | |  |
| *Missing* | 1388 | 39 | | 242 | 35 | 437 | | 28 | 455 | 31 | | | 2438 | | 32 | | 2900 | | 62 | |  |
| **At 10 years of age** |  |  | |  |  |  | |  |  |  | | |  | |  | |  | |  | |  |
| **HAZ, median (IQR)** | -0.35  (-1.13 ; 0.41) | | | -1.05  (-1.87 ;-0.22) | | -1.80  (-2.54 ; -1.07) | | | -1.37  (-2.14 ; -0.61) | | | -1.72  (-2.49 ; -0.99) | | | | -1.59  (2.34 ; -0.92) | | | | <0.001 | |
| **Severity of stunting, n %** |  |  | |  |  |  | |  |  |  | | |  | |  | |  | |  | | <0.001 |
| None | 2195 | 62 | | 342 | 49 | 718 | | 45 | 510 | 35 | | | 2367 | | 31 | | 1159 | | 25 | |  |
| Moderate | 149 | 4 | | 71 | 10 | 374 | | 24 | 169 | 12 | | | 1143 | | 15 | | 468 | | 10 | |  |
| Severe | 39 | 1 | | 22 | 3 | 171 | | 11 | 47 | 3 | | | 516 | | 7 | | 180 | | 4 | |  |
| *Missing* | 1184 | 33 | | 266 | 38 | 323 | | 20 | 725 | 50 | | | 3579 | | 47 | | 2844 | | 61 | |  |
| **BAZ, median (IQR)** | 0.25  (-0.47 ; 1.06) | | | -0.28  (-0.94 ; 0.37) | | -0.84  (-1.47 ; -0.16) | | | -0.85  (-1.54 ; -0.21) | | | -0.65  (-1.31 ; -0.03) | | | | -0.41  (-1.06 ; 0.25) | | | | <0.001 | |
| **Severity of wasting, n %** |  |  | |  |  |  | |  |  |  | | |  | |  | |  | |  | | <0.001 |
| None | 2335 | 65 | | 417 | 59 | 1109 | | 70 | 584 | 40 | | | 3579 | | 47 | | 1684 | | 36 | |  |
| Moderate | 33 | 1 | | 10 | 1 | 115 | | 7 | 80 | 6 | | | 289 | | 4 | | 90 | | 2 | |  |
| Severe | 7 | 0 | | 4 | 1 | 32 | | 2 | 24 | 2 | | | 83 | | 1 | | 24 | | 1 | |  |
| *Missing* | 1192 | 33 | | 270 | 39 | 330 | | 21 | 763 | 53 | | | 3654 | | 48 | | 2849 | | 61 | |  |
| **CD4 count, median (IQR)** | 750  (523 ; 1010) | | | 683  (438 ; 960) | | 779  (557 ; 1014) | | | 678  (452 ; 950) | | | 704  (464 ; 981) | | | | 755  (518 ; 1030) | | | | <0.001 | |
| **Immunodeficiency level, n %** |  |  | |  |  |  | |  |  |  | | |  | |  | |  | |  | | <0.001 |
| None | 2294 | 64 | | 374 | 53 | 1127 | | 71 | 768 | 53 | | | 3923 | | 52 | | 2302 | | 50 | |  |
| Moderate | 325 | 9 | | 73 | 10 | 140 | | 9 | 155 | 11 | | | 767 | | 10 | | 350 | | 8 | |  |
| Severe | 355 | 10 | | 92 | 13 | 134 | | 8 | 172 | 12 | | | 816 | | 11 | | 352 | | 8 | |  |
|  | 593 | 17 | | 162 | 23 | 185 | | 12 | 356 | 25 | | | 2099 | | 28 | | 1643 | | 35 | |  |
| **Follow-up and cohort profile** |  |  | |  |  |  | |  |  |  | | |  | |  | |  | |  | |  |
| **Year of birth, n %** |  | | |  | |  | | |  | | |  | | | |  | | | | <0.001 | |
| <1996 | 2039 | | 57 | 187 | 27 | 132 | 8 | | 89 | | 6 | 100 | | 1 | | 211 | | 5 | |  | |
| [1996-2000[ | 888 | | 25 | 302 | 43 | 531 | 33 | | 680 | | 47 | 2461 | | 32 | | 1882 | | 40 | |  | |
| >2000 | 640 | | 18 | 212 | 30 | 923 | 58 | | 682 | | 47 | 5044 | | 66 | | 2554 | | 55 | |  | |
| **Age at last visit, median (IQR)** | 15.3  (12.9 ; 17.6) | | | 15.0  (12.9 ; 17.5) | | 13.1  (11.5 ; 14.9) | | | 12.4  (11.3 ; 14.2) | | | 12.5  (11.4 ; 14.0) | | | | 12.7  (11.4 ; 14.5) | | | | <0.001 | |
| **Status at last visit** |  |  | |  |  |  | |  |  |  | | |  | |  | |  | |  | |  |
| Alive, in follow-up | 1741 | 49 | | 511 | 73 | 1316 | | 83 | 1237 | 85 | | | 4564 | | 60 | | 3018 | | 65 | | <0.001 |
| Lost-to-Follow-up‡ | 1097 | 31 | | 92 | 13 | 164 | | 10 | 136 | 9 | | | 2204 | | 29 | | 616 | | 13 | |  |
| Transferred | 685 | 19 | | 65 | 9 | 78 | | 5 | 42 | 3 | | | 690 | | 9 | | 978 | | 21 | |  |
| Dead | 44 | 1 | | 33 | 5 | 28 | | 2 | 36 | 2 | | | 147 | | 2 | | 35 | | 1 | |  |
| **Patient data contribution, n obs %** |  |  | |  |  |  | |  |  |  | | |  | |  | |  | |  | | <0.001 |
| Between 10 and 14 years | 48,596 | 34 | | 6672 | 5 | 11,287 | | 8 | 8982 | 6 | | | 47,095 | | 33 | | 21 ,916 | | 15 | |  |
| Between 15 and 17 years | 11,317 | 60 | | 1371 | 7 | 968 | | 5 | 647 | 3 | | | 2625 | | 14 | | 2061 | | 11 | |  |
| ART: Antiretroviral therapy, BAZ: BMI-for-age Z-score, IQR: Interquartile Range, HAZ: Height-for-age Z-score, WHZ: Weight-for-Height Z-score, obs=observations.  *Chi-square or Kruskal-Wallis tests. † WHO 2006 guidelines. ‡ Defined as no contact for more than 365 days or documented loss-to-follow-up. | | | | | | | | | | | | | | | | | | | | | |


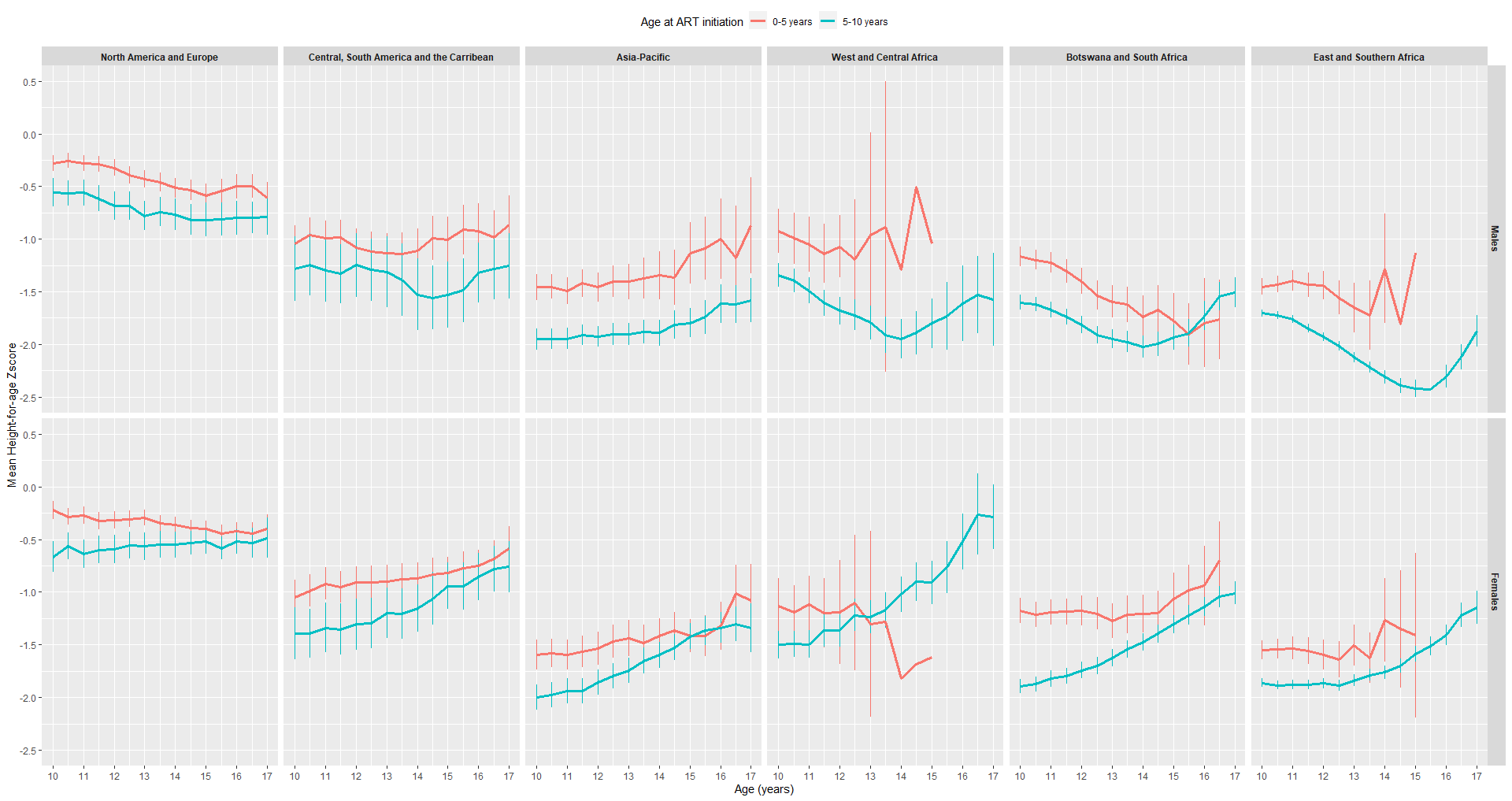


**Supplementary Figure 1: Adjusted estimated mean Height-for-age Z-scores for adolescents living with perinatally acquired HIV, stratified by age at ART initiation, according to sex and regions. CIPHER global cohort collaboration, 1994-2015**


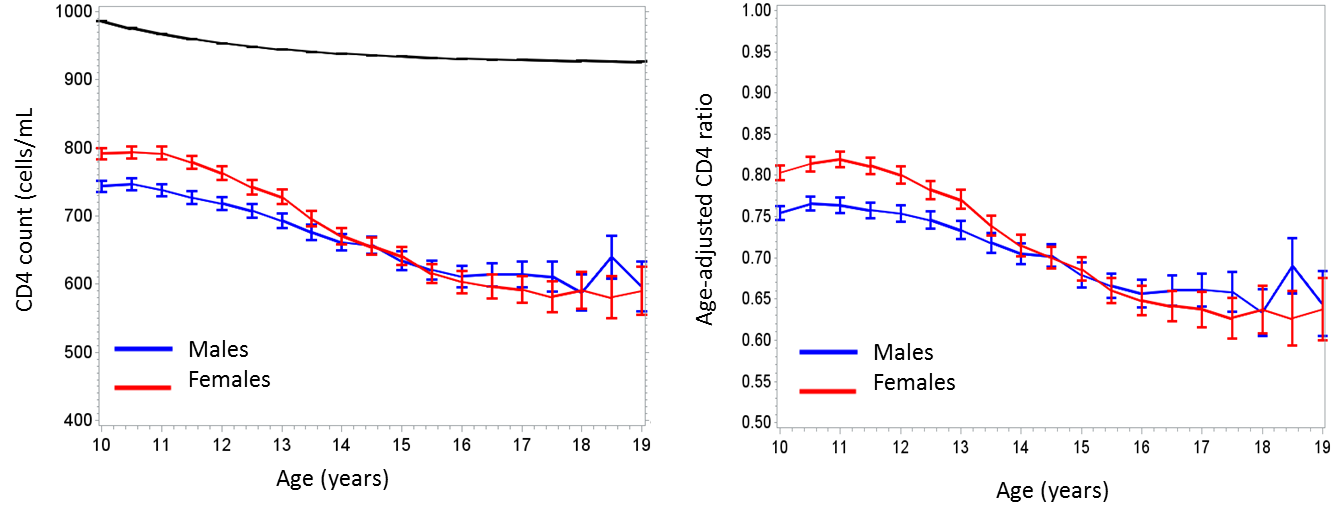


**Supplementary Figure 2: Mean CD4 evolution during adolescence for adolescents living with perinatally acquired HIV, stratified by sex, expressed in cells/mm^3^ (left) and in age-adjusted ratio (right). CIPHER global cohort collaboration, 1994-2015**


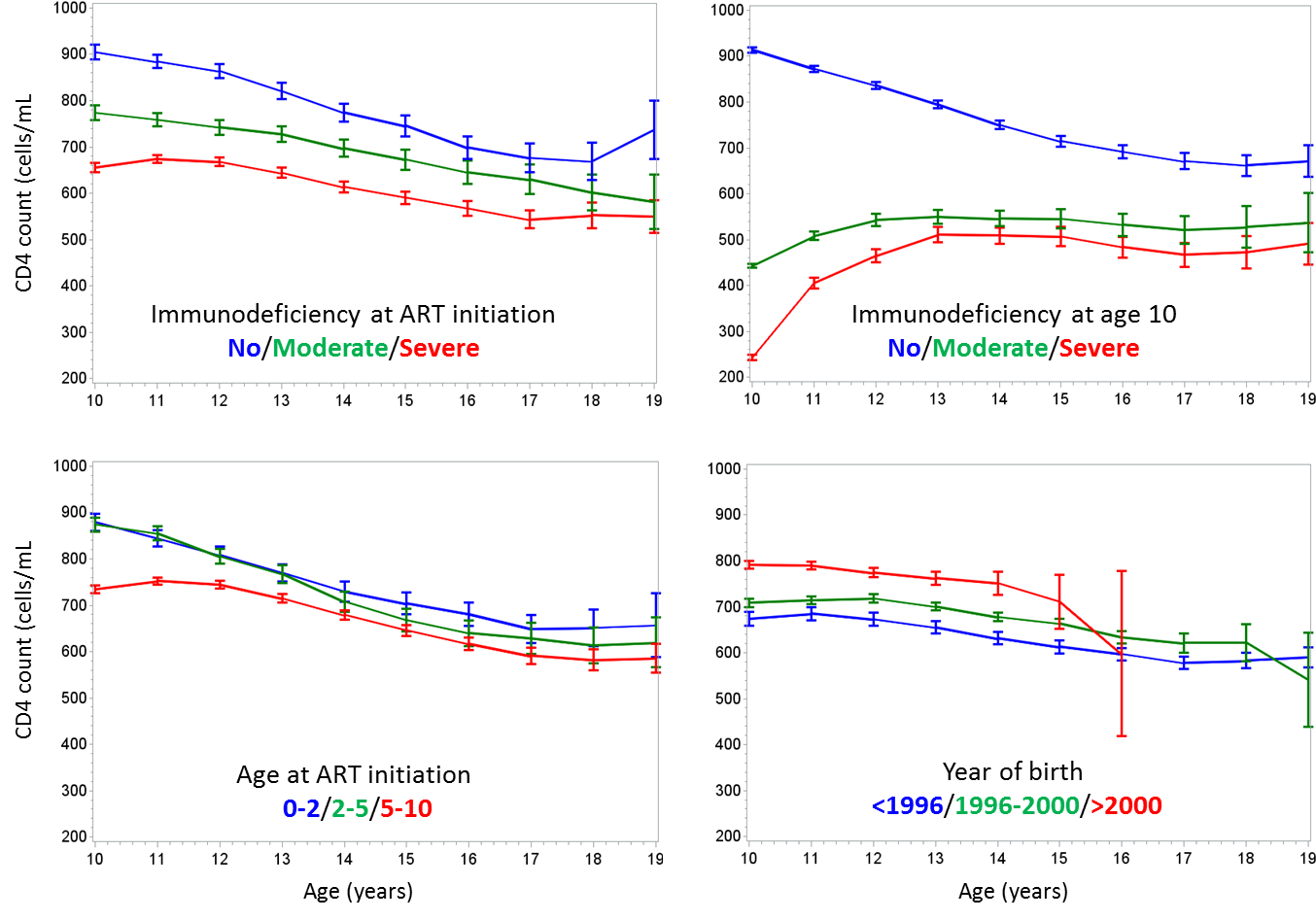


**Supplementary Figure 3: Mean CD4 evolution during adolescence for adolescents living with perinatally acquired HIV, stratified by immunodeficiency at ART initiation and at age 10, as well as by age at ART initiation and year of birth. CIPHER global cohort collaboration, 1994-2015**

**Supplementary results: Joint modeling of growth and immunology, illustrations for males, for two regions (North America & Europe, East and Southern Africa excluding Botswana and South Africa)**

The main steps of the analyses were:

- We identified, for each outcome, profiles of population following similar trajectories. The number of trajectories was determined by taking into account both the AIC and different proportion of patients included in each trajectory. Most of the models for each region identified three main trajectories (low, median and high). Trajectory curves are shown below for each region, by sex, for CD4 and growth between 10 and 19 years of age.
- After having determined trajectory profiles, we fit a joint model giving the contribution of each identified trajectory from one outcome to the identified trajectory from the other outcome. For example, among those having a low CD4 trajectory (low at baseline and remaining low during adolescence), the joint model could tell us how much they were contributing to the low HAZ trajectory, to the median one etc. The results of each group are shown Tables A and B. A Chi square test was conducted to determine if the contribution for each outcome differed according to the trajectory. (i.e., are the three different profiles of growth evolution identified distributed equally into the three different profiles identified for CD4 evolution?)
- To go further, we also conducted a multi-trajectory analysis. This time, the profiles were not determined with only one outcome but using both outcomes. This gave us 3 different profiles having a specific trajectory for CD4, associated with a specific trajectory for growth. This approach could help us to retain more information and to see graphically if CD4 and HAZ evolved concomitantly during adolescence.

# North America and Europe – Males, N=1656

**
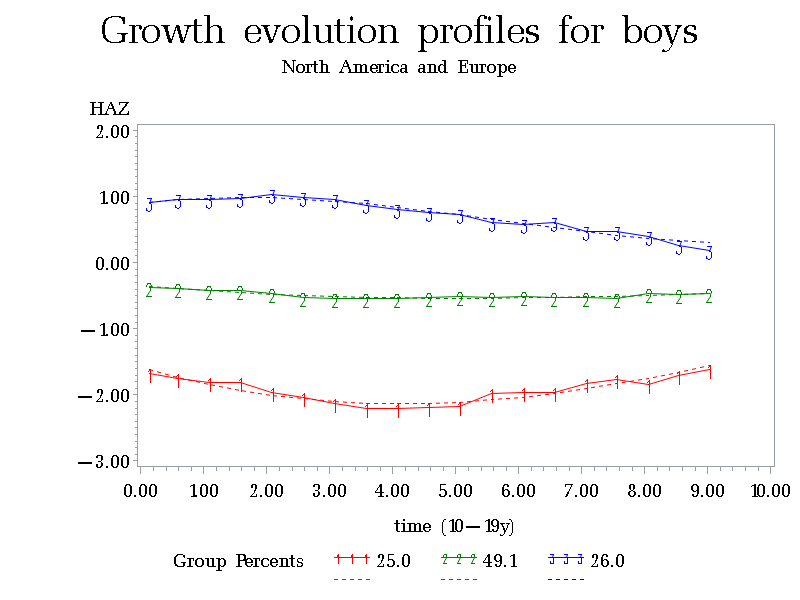

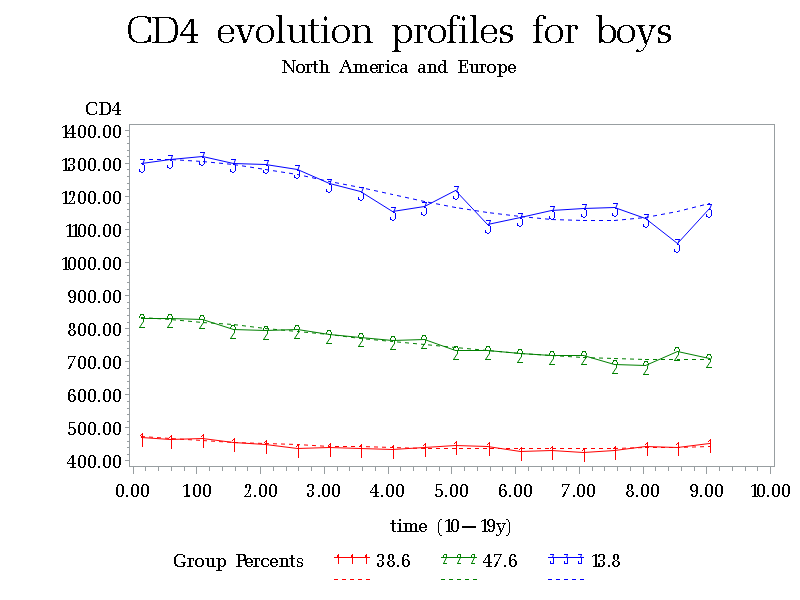
Group-based trajectory models for CD4 and Growth (figures) and joint trajectory model estimates (table)**

| **North America and Europe - Males** | | **Group 1 (Low)** | **Group 2 (Medium)** | **Group 3 (High)** |
| --- | --- | --- | --- | --- |
| **Joint** probabilities of HAZ and CD4 (%) | | | | |
|  |  | CD4 | | |
| **Group 1 (Low)** | HAZ | 11.0 | 9.9 | 3.6 |
| **Group 2 (Medium)** |  | 17.0 | 25.3 | 6.9 |
| **Group 3 (High)** |  | 9.7 | 12.8 | 3.8 |
| Probabilities of **growth** trajectory membership conditional of **CD4** trajectory membership | | | | |
|  | p=0.712 | CD4 | | |
| **Group 1 (Low)** | HAZ | 29.1 | 20.5 | 25.1 |
| **Group 2 (Medium)** |  | 45.1 | 52.8 | 48.2 |
| **Group 3 (High)** |  | 25.7 | 26.7 | 26.7 |
| Probabilities of **CD4** trajectory membership conditional of **growth** trajectory membership | | | | |
|  | p=0.563 | CD4 | | |
| **Group 1 (Low)** | HAZ | 45.0 | 40.3 | 14.6 |
| **Group 2 (Medium)** |  | 34.6 | 51.4 | 13.9 |
| **Group 3 (High)** |  | 37.0 | 48.6 | 14.4 |

**Group-based multi-trajectory model**

# East and Southern Africa (except Botswana and South Africa) – Males, N=5642


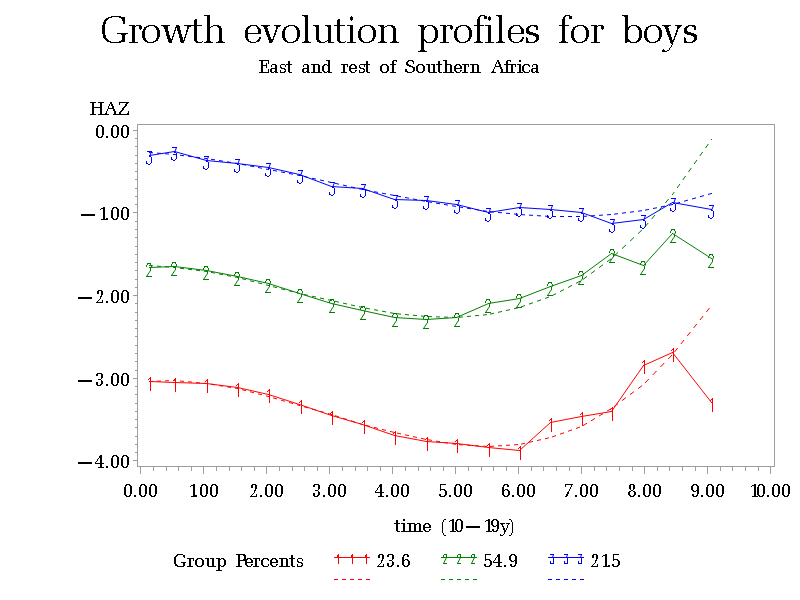

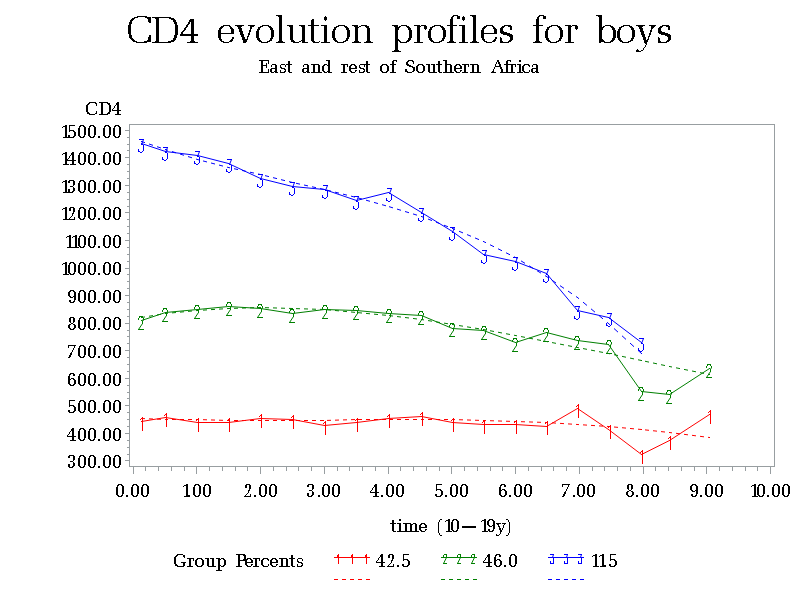
**Group-based trajectory models for CD4 and Growth (figures) and joint trajectory model estimates (table)**

| **East and rest of Southern Africa- Males** | | **Group 1 (Low)** | **Group 2 (Medium)** | **Group 3 (High)** |
| --- | --- | --- | --- | --- |
| **Joint** probabilities of HAZ and CD4 (%) | | | | |
|  |  | CD4 | | |
| **Group 1 (Low)** | HAZ | 11.6 | 9.5 | 2.8 |
| **Group 2 (Medium)** |  | 22.5 | 25.3 | 6.5 |
| **Group 3 (High)** |  | 9.8 | 10.1 | 1.8 |
| Probabilities of **growth** trajectory membership conditional of **CD4** trajectory membership | | | | |
|  | p=0.676 | CD4 | | |
| **Group 1 (Low)** | HAZ | 26.4 | 21.2 | 25.5 |
| **Group 2 (Medium)** |  | 51.2 | 56.3 | 58.2 |
| **Group 3 (High)** |  | 22.3 | 22.5 | 16.3 |
| Probabilities of **CD4** trajectory membership conditional of **growth** trajectory membership | | | | |
|  | p=0.734 | CD4 | | |
| **Group 1 (Low)** | HAZ | 48.4 | 39.7 | 11.9 |
| **Group 2 (Medium)** |  | 41.5 | 46.6 | 12.0 |
| **Group 3 (High)** |  | 45.2 | 46.4 | 8.4 |

**Group-based multi-trajectory model**
