# Supplementary material for: Growth and CD4 patterns of adolescents living with perinatally acquired HIV worldwide, a CIPHER cohort collaboration analysis
Source: J Int AIDS Soc. 2022 Mar 7;25(3):e25871. doi: 10.1002/jia2.25871 (PMC8901148; doi:10.1002/jia2.25871)
Supplement: Supplementary file 2 — Data S1: Full list of acknowledgments for the CIPHER Global Cohort Collaboration [file JIA2-25-e25871-s001.docx]

**Full list of acknowledgements – CIPHER Global Cohort Collaboration - Growth and CD4 patterns of adolescents living with perinatally acquired HIV worldwide, a CIPHER cohort collaboration analysis – January 2022**

**CIPHER Executive Committee:** Linda-Gail Bekker (Desmond Tutu HIV Centre, University of Cape Town, South Africa), Lynne Mofenson (Elizabeth Glaser Pediatric AIDS Foundation, USA), Marissa Vicari (International AIDS Society, Switzerland),

Shaffiq Essajee (UNICEF), Martina Penazzato (World Health Organization, Switzerland).

**CIPHER Steering Committee:** Elaine Abrams (ICAP-Columbia University, Mailman School of Public Health, USA), Mary Paul (Baylor International Pediatric AIDS Initiative, USA),

Jihane Ben-Farhat (Médecins Sans Frontières), Russell Van Dyke (Tulane University Health Sciences Center, USA), Rachel Vreeman (Icahn School of Medicine at Mount Sinai, USA), Lynne Mofenson (Elizabeth Glaser Pediatric AIDS Foundation), Marissa Vicari (International AIDS Society), George Seage III (Harvard T. H. Chan School of Public Health, USA), Linda-Gail Bekker (Desmond Tutu HIV Centre, University of Cape Town), Shaffiq Essajee (UNICEF, USA), Diana Gibb (MRC Clinical Trials Unit at UCL, UK).

**Project Oversight Group:** Intira Jeannie Collins (MRC Clinical Trials Unit at UCL, UK), Kara Wools-Kaloustian (Indiana University School of Medicine, USA), Mary-Ann Davies (University of Cape Town, South Africa), Valériane Leroy (INSERM, Université Victor Segalen Bordeaux, France), Ruth Goodall (MRC Clinical Trials Unit at UCL, UK), Kunjal Patel (Harvard T. H. Chan School of Public Health, USA), Colette Smith (MRC Clinical Trials Unit at UCL, UK), Rachel Vreeman (Icahn School of Medicine at Mount Sinai, USA), Amy Slogrove (Stellenbosch University, Worcester, South Africa), Kathleen Powis (Harvard T. H. Chan School of Public Health, USA), Paige Williams (Harvard T. H. Chan School of Public Health, USA), Julie Jesson (Université Toulouse), Siobhan Crichton (MRC Clinical Trials Unit at UCL, UK), George Seage III (Harvard T. H. Chan School of Public Health, USA).

**The following sites, their personnel and patients are acknowledged for their contribution to the participating networks that made the CIPHER Cohort Collaboration Adolescent Project possible:**

**BIPAI**: Baylor College of Medicine Children's Foundation Lesotho, Lineo Thahane; Baylor College of Medicine Children's Foundation Malawi, Peter N. Kazembe; Baylor College of Medicine Children’s Foundation-eSwatini, Bhekumusa Lukhele; Baylor College of Medicine Children's Foundation-Tanzania, Lumumba Mwita; Baylor College of Medicine Children's Foundation – Uganda, Adeodata Kekitiinwa-Rukyalekere; Botswana-Baylor Children's Clinical Centre of Excellence, Mogomotsi S. Matshaba; Baylor International Pediatric AIDS Initiative at Texas Children’s Hospital Senior Vice President for Research, Nancy Calles; Baylor International Pediatric AIDS Initiative at Texas Children’s Hospital Founder, Mark Kline.

**EPPICC: *Belgium:*** Hospital St Pierre Cohort, Brussels: Tessa Goetghebuer, MD, PhD; Marc Hainaut, MD PhD; Evelyne Van der Kelen, Research nurse; Marc Delforge, data manager. ***France:*** French Perinatal Cohort Study/Enquête Périnatale Française, ANRS EPF-CO10. Coordinating center, INSERM U1018, team 4: Josiane Warszawski, Jerome Le Chenadec, Elisa Ramos, Olivia Dialla, Thierry Wack, Corine Laurent, Lamya Ait si Selmi, Isabelle Leymarie, Fazia Ait Benali, Maud Brossard, Leila Boufassa. Participating sites: Hôpital Louis Mourier, Colombes, Dr Corinne Floch-Tudal; Groupe Hospitalier Cochin Tarnier Port-Royal, Paris, Dr Ghislaine Firtion; Centre Hospitalier Intercommunal, Creteil, Dr Isabelle Hau; Centre Hospitalier Général, Villeneuve Saint Georges, Dr Anne Chace; Centre Hospitalier Général- Hôpital Delafontaine, Saint-Denis, Dr Pascal Bolot; Groupe Hospitalier Necker, Paris, Pr Stéphane Blanche; Centre hospitalier Francilien Sud, Corbeil Essonne, Dr Michèle Granier; Hôpital Antoine Béclère, Clamart, Pr Philippe Labrune; Hôpital Jean Verdier, Bondy, Dr Eric Lachassine; Hôpital Trousseau, Paris, Dr Catherine Dollfus; Hôpital Robert Debré, Paris, Dr Martine Levine; Hôpital Bicêtre, Le Kremlin Bicëtre, Dr Corinne Fourcade; Centre Hospitalier Intercommunal, Montreuil, Dr Brigitte Heller- Roussin; Centre Hospitalier Pellegrin, Bordeaux, Dr Camille Runel-Belliard; CHU Paule de Viguier, Toulouse, Dr Joëlle Tricoire; CHU Hôpital de l'Archet II, Nice, Dr Fabrice Monpoux; Groupe Hospitalier de la Timone, Marseille; CHU Hôpital Jean Minjoz, Besancon, Dr Catherine Chirouze; CHU Nantes Hotel Dieu, Nantes, Dr Véronique Reliquet; CHU Caen, Caen, Pr Jacques Brouard; Institut d’Hématologie et Oncologie Pédiatrique, Lyon, Dr Kamila Kebaili; CHU Angers, Angers, Dr Pascale Fialaire; CHR Arnaud de Villeneuve, Montpellier, Dr Muriel Lalande; CHR Jeanne de Flandres, Lille, Dr Françoise Mazingue; Hôpital Civil, Strasbourg, Dr Maria Luisa Partisani. ***Germany:*** German Paediatric & Adolescent HIV Cohort (GEPIC): Dr Christoph Königs, Dr Stephan Schultze-Strasser. German clinical centers: Hannover Medical School, Dr. U. Baumann; Pediatric Hospital Krefeld, Dr. T. Niehues; University Hospital Düsseldorf, Dr. J. Neubert; University Hospital Hamburg, Dr. R. Kobbe; Charite Berlin, Dr. C. Feiterna-Sperling; University Hospital Frankfurt, Dr. C. Königs; University Hospital Mannheim, Dr. B. Buchholz; Munich University Hospital, Dr. G. Notheis.

***Greece:*** Greek cohort: Vana Spoulou. ***Italy:*** Italian Register for HIV infection in Children. Coordinators: Elena Chiappini, Maurizio de Martino, Luisa Galli, (Florence), Pier Angelo Tovo, Clara Gabiano (Turin). Participants: Osimani Patrizia (Ancona), Domenico Larovere (Bari), Maurizio Ruggeri (Bergamo), Giacomo Faldella, Francesco Baldi (Bologna) Raffaele Badolato (Brescia), Carlotta Montagnani, Elisabetta Venturini, Leila Bianchi, Catiuscia Lisi (Florence), Antonio Di Biagio, Lucia Taramasso (Genua), Vania Giacomet, Paola Erba, Susanna Esposito, Rita Lipreri, Filippo Salvini, Claudia Tagliabue (Milan), Monica Cellini (Modena), Eugenia Bruzzese, Andrea Lo Vecchio (Naples), Osvalda Rampon, Daniele Donà (Padua), Amelia Romano (Palermo), Icilio Dodi (Parma), Anna Maccabruni (Pavia), Rita Consolini (Pisa), Stefania Bernardi, Hyppolite Tchidjou Kuekou, Orazio Genovese (Rome), Paolina Olmeo (Sassari), Letizia Cristiano (Taranto), Antonio Mazza (Trento), Silvia Garazzino (Turin), Antonio Pellegatta (Varese). ***Netherlands:*** The ATHENA database is maintained by Stichting HIV Monitoring and supported by a grant from the Dutch Ministry of Health, Welfare and Sport through the Centre for Infectious Disease Control of the National Institute for Public Health and the Environment. CLINICAL CENTRES (PAEDIATRIC CARE): Emma Kinderziekenhuis (Amsterdam UMC, AMC site): HIV treating physicians: M. van der Kuip, D. Pajkrt H.J. Scherpbier. HIV nurse consultants: C. de Boer, A.M. Weijsenfeld. HIV clinical virologists/chemists: S. Jurriaans, N.K.T. Back, H.L. Zaaijer, B. Berkhout, M.T.E. Cornelissen, C.J. Schinkel, K.C. Wolthers. Erasmus MC–Sophia, Rotterdam: HIV treating physicians: P.L.A. Fraaij, A.M.C. van Rossum, C.L. Vermont. HIV nurse consultants: L.C. van der Knaap, E. Visser, J van der Waal. HIV clinical virologists/chemists: C.A.B. Boucher, M.P.G Koopmans, J.J.A van Kampen. Radboudumc, Nijmegen: HIV treating physicians: S.S.V. Henriet, M. K. van Aerde. HIV nurse consultants: R. Strik-Albers. HIV clinical virologists/chemists: J. Rahamat-Langendoen, F.F. Stelma. HIV clinical pharmacology consultant: D. Burger Universitair Medisch Centrum Groningen/Beatrix Kinderziekenhuis, Groningen: HIV treating physicians: E.H. Schölvinck, A.R. Verhage. HIV nurse consultants: H. de Groot-de Jonge. HIV clinical virologists/chemists: H.G.M. Niesters, C.C. van Leer-Buter, M. Knoester. Wilhelmina Kinderziekenhuis, UMC Utrecht, Utrecht: HIV treating physicians: L.J. Bont, S.P.M. Geelen, Y.G.T. Loeffen, T.F.W. Wolfs. HIV nurse consultants: N. Nauta. HIV clinical virologists/chemists: R. Schuurman, L.M Hofstra, A.M.J. Wensing. COORDINATING CENTRE: Director: P. Reiss. Deputy director: S. Zaheri. Data analysis: A.C. Boyd, D.O. Bezemer, A.I. van Sighem, C. Smit, F.W.M.N. Wit. Data management and quality control: M.M.J. Hillebregt, T.J. Woudstra. Data monitoring: D. Bergsma, L. van de Sande, T. Rutkens, S. van der Vliet, K.J Lelivelt, A Scheijgrond. Data collection: L. de Groot, M. van den Akker, Y. Bakker, A. El Berkaoui, M. Bezemer, N. Brétin, E. Djoechro, M. Groters, E. Kruijne, K.J. Lelivelt, C. Lodewijk, E. Lucas, L. Munjishvili, F. Paling, B. Peeck, C. Ree, R. Regtop, Y. Ruijs, M. Schoorl, P. Schnörr, E. Tuijn, L. Veenenberg, K.M. Visser, E.C. Witte. Patient registration: Y. Ruijs Clinical centres (paediatric care) Emma Kinderziekenhuis, Academic Medical Centre of the University of Amsterdam: HIV treating physicians: D. Pajkrt, H.J. Scherpbier. HIV nurse consultants: A.M. Weijsenfeld, C.G. de Boer. HIV clinical virologists/chemists: S. Jurriaans, N.K.T. Back, H.L. Zaaijer, B. Berkhout, M.T.E. Cornelissen, C.J. Schinkel, K.C.Wolthers. Erasmus MC–Sophia, Rotterdam: HIV treating physicians: P.L.A. Fraaij, A.M.C. van Rossum. HIV nurse consultants: L.C. van der Knaap, E.G. Visser. HIV clinical virologists/chemists: C.A.B. Boucher, M.P.G Koopmans, J.J.A van Kampen, S.D. Pas. Radboudumc, Nijmegen: HIV treating physicians: S.S.V. Henriet, M. van de Flier, K. van Aerde. HIV nurse consultants: R. Strik-Albers. HIV clinical virologists/chemists: J. Rahamat-Langendoen, F.F. Stelma. Universitair Medisch Centrum Groningen, Groningen: HIV treating physicians: E. H. Schölvinck. HIV nurse consultants: H. de Groot-de Jonge. HIV clinical virologists/chemists: H.G.M. Niesters, C.C. van Leer-Buter, M. Knoester. Wilhelmina Kinderziekenhuis, UMCU, Utrecht: HIV treating physicians: L.J. Bont, S.P.M. Geelen, T.F.W. Wolfs. HIV nurse consultants: N. Nauta. HIV clinical virologists/chemists: C.W. Ang, R. van Houdt, A.M. Pettersson, C.M.J.E. Vandenbroucke-Grauls. Coordinating Centre: Director: P. Reiss. Data analysis: D.O. Bezemer, A.I. van Sighem, C. Smit, F.W.M.N. Wit, T.S. Boender. Data management and quality control: S. Zaheri, M. Hillebregt, A. de Jong. Data monitoring: D. Bergsma, S. Grivell, A. Jansen, M. Raethke, R. Meijering. Data collection: L. de Groot, M. van den Akker, Y. Bakker, E. Claessen, A. El Berkaoui, J. Koops, E. Kruijne, C. Lodewijk, L. Munjishvili, B. Peeck, C. Ree, R. Regtop, Y. Ruijs, T. Rutkens, M. Schoorl, A. Timmerman, E. Tuijn, L. Veenenberg, S. van der Vliet, A. Wisse, T. Woudstra. Patient registration: B. Tuk. **Poland**: Polish paediatric cohort: Head of the team: Prof Magdalena Marczyńska. Members of the team: Jolanta Popielska; Maria Pokorska-Śpiewak; Agnieszka Ołdakowska; Konrad Zawadka; Urszula Coupland. Administration assistant: Małgorzata Doroba, Medical University of Warsaw, Poland, Department of Children’s Infectious Diseases; Hospital of Infectious Diseases in Warsaw, Poland. **Portugal**: Centro Hospitalar do Porto:Laura Marques, Carla Teixeira, Alexandre Fernandes, Hospital de Santa Maria/CHLN: Filipa Prata. Romania: "Victor Babes" Hospital Cohort, Bucharest: Dr Luminita Ene. **Russia**: Federal State-owned Institution "Republican Clinical Infectious Diseases Hospital" of the Ministry of Health of the Russian Federation, St Petersburg: Liubov Okhonskaia, Evgeny Voronin, Milana Miloenko, Svetlana Labutina. **Spain**: CoRISPE-cat, Catalonia: financial support for CoRISPE-cat was provided by the Instituto de Salud Carlos III through the Red Temática de Investigación Cooperativa en Sida. Members: Hospital Universitari Vall d’Hebron, Barcelona (Pere Soler-Palacín, Maria Antoinette Frick and Santiago Pérez-Hoyos (statistician), Hospital Universitari del Mar, Barcelona (Antonio Mur, Núria López), Hospital Universitari Germans Trias i Pujol, Badalona (María Méndez), Hospital Universitari JosepTrueta, Girona (Lluís Mayol), Hospital Universitari Arnau de Vilanova, Lleida (Teresa Vallmanya), Hospital Universitari Joan XXIII, Tarragona (Olga Calavia), Consorci Sanitari del Maresme, Mataró (Lourdes García), Hospital General de Granollers (Maite Coll), Corporació Sanitària Parc Taulí, Sabadell (Valentí Pineda), Hospital Universitari Sant Joan, Reus (Neus Rius), Fundació Althaia, Manresa (Núria Rovira), Hospital Son Espases, Mallorca (Joaquín Dueñas) and Hospital Sant Joan de Déu, Esplugues (Clàudia Fortuny, Anna Gamell, Antoni Noguera-Julian). CoRISPE-S and Madrid cohort collaborating members: María José Mellado, Luis Escosa, Milagros García Hortelano, Talía Sainz (Hospital Universitario La Paz, Madrid); Pablo Rojo, Daniel Blázquez, Luis Prieto-Tato, Cristina Epalza (Hospital Universitario Doce de Octubre, Madrid); José Tomás Ramos (Hospital Clínico San Carlos, Madrid); Sara Guillén (Hospital Universitario de Getafe, Madrid); María Luisa Navarro, Jesús Saavedra, Mar Santos, Begoña Santiago, Santiago Jimenez de Ory, Itzíar Carrasco, Mª Angeles Muñoz-Fernández (Hospital Universitario Gregorio Marañón, Madrid); Miguel Ángel Roa (Hospital Universitario de Móstoles, Madrid); María Penín (Hospital Universitario Príncipe de Asturias de Alcalá de Henares, Madrid); Jorge Martínez (Hospital Infantil Universitario Niño Jesús, Madrid); Katie Badillo (Hospital Universitario de Torrejón, Madrid); Eider Oñate (Hospital Universitario Donostia, Guipúzcoa); Itziar Pocheville (Hospital Universitario Cruces, Vizcaya); Elisa Garrote (Hospital Universitario Basurto, Vizcaya); Elena Colino (Hospital Insular Materno Infantil, Gran Canaria); Jorge Gómez Sirvent (Hospital Universitario Virgen de la Candelaria, Tenerife); Mónica Garzón, Vicente Román (Hospital General, Lanzarote); Raquel Angulo (Hospital de Poniente de El Ejido, Almería); Olaf Neth, Lola Falcón (Hospital Universitario Virgen del Rocío, Sevilla); Pedro Terol (Hospital Universitario Virgen de la Macarena, Sevilla); Juan Luis Santos (Hospital Universitario Virgen de las Nieves, Granada); David Moreno (Hospital Regional Universitario Carlos Haya, Málaga); Francisco Lendínez (Complejo Hospitalario Torrecárdenas, Almería); Estrella Peromingo (Hospital Universitario Puerta del Mar, Cádiz); José Uberos (Hospital Clínico San Cecilio, Granada); Beatriz Ruiz (Hospital Universitario Reina Sofía de Córdoba); Ana Grande (Complejo Hospitalario Universitario Infanta Cristina, Badajoz); Francisco José Romero (Complejo Hospitalario, Cáceres); Carlos Pérez (Hospital de Cabueñes, Asturias); Miguel Lillo (Complejo Hospitalario Universitario, Albacete); Begoña Losada (Hospital Virgen de la Salud, Toledo); Mercedes Herranz (Hospital Virgen del Camino, Navarra); Matilde Bustillo (Hospital Universitario Miguel Servet, Zaragoza); Pilar Collado (Hospital Clínico Universitario Lozano Blesa, Zaragoza); José Antonio Couceiro (Complejo Hospitalario Universitario, Pontevedra); Leticia Vila (Complejo Hospitalario Universitario, La Coruña); Consuelo Calviño (Hospital Universitario Lucus Augusti, Lugo); Ana Isabel Piqueras, Manuel Oltra (Hospital Universitario La Fe, Valencia); César Gavilán (Hospital Universitario de San Juan de Alicante, Alicante); Elena Montesinos (Hospital General Universitario, Valencia); Marta Dapena (Hospital General, Castellón); Cristina Álvarez, Beatriz Jiménez (Hospital Universitario Marqués de Valdecilla, Cantabria); Ana Gloria Andrés (Complejo Hospitalario, León); Víctor Marugán, Carlos Ochoa (Complejo Hospitalario, Zamora); Santiago Alfayate, Ana Isabel Menasalvas (Hospital Universitario Virgen de la Arrixaca, Murcia); Yolanda Ruiz del Prado (Complejo Hospitalario San Millán-San Pedro, la Rioja) and Paediatric HIV-BioBank integrated in the Spanish AIDS Research Network and collaborating Centers. Financial support for CoRISpeS and Madrid Cohort was provided by the Instituto de Salud Carlos III through the Red Tematica de Investigacion Cooperativa en Sida (RED-RIS) project as part of the Plan R+D+I and cofinanced by ISCIII- Subdireccion General de Evaluación and Fondo Europeo de Desarrollo Regional (FEDER). María José Mellado, Luis Escosa, Milagros García Hortelano, Talía Sainz (Hospital Universitario La Paz, Madrid); María Isabel González- Tomé, Pablo Rojo, Luis Prieto-Tato, Cristina Epalza (Hospital Universitario Doce de Octubre, Madrid); José Tomás Ramos (Hospital Clínico San Carlos, Madrid); Sara Guillén (Hospital Universitario de Getafe, Madrid); María Luisa Navarro, Jesús Saavedra, Mar Santos, Mª Angeles Muñoz, Begoña Santiago, Santiago Jimenez de Ory, Itziar Carrasco (Hospital Universitario Gregorio Marañón, Madrid); Miguel Ángel Roa (Hospital Universitario de Móstoles, Madrid); José Beceiro, María Penín (Hospital Universitario Príncipe de Asturias de Alcalá de Henares, Madrid); Jorge Martínez (Hospital Infantil Universitario Niño Jesús, Madrid); Katie Badillo (Hospital de Torrejón, Madrid); Miren Apilanez (Hospital de Donostia, Guipúzcoa); Itziar Pocheville (Hospital de Cruces, Vizcaya); Elisa Garrote (Hospital de Basurto, Vizcaya); Elena Colino (Hospital Insular Materno Infantil, Gran Canaria); Jorge Gómez Sirvent (Hospital Universitario Virgen de la Candelaria, Tenerife); Mónica Garzón, Vicente Román (Hospital General, Lanzarote); Abián Montesdeoca, Mercedes Mateo (Complejo Hospitalario Universitario, Tenerife); Raquel Angulo (Hospital de Poniente de El Ejido, Almería); Olaf Neth, Lola Falcón (Hospital Universitario Virgen del Rocio, Sevilla); Pedro Terol (Hospital Universitario Virgen de la Macarena, Sevilla); Juan Luis Santos (Hospital Universitario Virgen de las Nieves, Granada); David Moreno (Hospital Regional Universitario Carlos Haya, Málaga); Francisco Lendínez (Complejo Hospitalario Torrecárdenas, Almería); Estrella Peromingo, María Montero (Hospital Universitario Puerta del Mar, Cádiz); José Uberos (Hospital Clínico San Cecilio, Granada); Digna Espinosa, Rocío Montiel, María del Carmen Gutiérrez Moro (Hospital La Línea de La Concepción, Cádiz); Beatriz Ruiz (Hospital Universitario Reina Sofía de Córdoba); Ana Grande (Complejo Hospitalario Universitario Infanta Cristina, Badajoz); Francisco José Romero (Complejo Hospitalario, Cáceres); Carlos Pérez (Hospital de Cabueñes, Asturias); María Martínez (Hospital Universitario Central, Asturias); Miguel Lillo (Complejo Hospitalario Universitario, Albacete); Begoña Losada (Hospital Virgen de la Salud, Toledo); Mercedes Herranz (Hospital Virgen del Camino, Navarra); Matilde Bustillo, Carmelo Guerrero (Hospital Universitario Miguel Servet, Zaragoza); Pilar Collado (Hospital Clínico Universitario Lozano Blesa, Zaragoza); María Jesús Oliván (Hospital San Jorge, Huesca); José Antonio Couceiro (Complejo Hospitalario Universitario, Pontevedra); Leticia Vila (Complejo Hospitalario Universitario, La Coruña); Consuelo Calviño (Hospital Universitario Lucus Augusti, Lugo); Ana Isabel Piqueras, Manuel Oltra (Hospital Universitario La Fe, Valencia); César Gavilán (Hospital Universitario de San Juan de Alicante, Alicante); Elena Montesinos (Hospital General Universitario, Valencia); Marta Dapena (Hospital General, Castellón); Enrique Jareño (Hospital Clínico Universitario, Valencia); Cristina Álvarez, Beatriz Jiménez (Hospital Universitario Marqués de Valdecilla, Cantabria); Ana Gloria Andrés (Complejo Hospitalario, León); Víctor Marugán, Carlos Ochoa (Complejo Hospitalario, Zamora); Elena Urbaneja (Hospital Clínico Universitario, Valladolid); Santiago Alfayate, Ana Isabel Menasalvas (Hospital Universitario Virgen de la Arrixaca, Murcia); Yolanda Ruiz del Prado (Complejo Hospitalario San Millán San Pedro, la Rioja) Funding: This work has been partially funded by the Fundación para la Investigación y Prevención de SIDA en España (FIPSE) (FIPSE 3608229/09 , FIPSE 240800/09, FIPSE 361910/10), Red Temática de Investigación en SIDA (RED RIS) supported by Instituto de Salud Carlos III (ISCIII) (RD12/0017/0035 and RD12/0017/0037), project as part of the Plan R+D+I and cofinanced by ISCIII- Subdirección General de Evaluación and Fondo Europeo de Desarrollo Regional (FEDER),Mutua Madrileña 2012/0077, Gilead Fellowship 2013/0071, FIS PI15/00694 ,CoRISpe (RED RIS RD06/0006/0035 y RD06/0006/0021). **Sweden**: Karolinska Institutet and University Hospital and Karolinska Institutet, Stockholm (Lars Naver, Erik Belfrage, Sandra Soeria-Atmadja, Vendela Hagås). **Switzerland**: Members of the Swiss HIV Cohort Study (SHCS) and the Swiss Mother and Child HIV Cohort Study (MoCHiV): Aebi-Popp K, Anagnostopoulos A, Battegay M, Baumann M, Bernasconi E, Böni J, Braun DL, Bucher HC, Calmy A, Cavassini M, Ciuffi A, Crisinel PA, Duppenthaler A, Dollenmaier G, Egger M, Elzi L, Fehr J, Fellay J, Francini K, Furrer H, Fux CA, Günthard HF (President of the SHCS), Haerry D (deputy of "Positive Council"), Hasse B, Hirsch HH, Hoffmann M, Hösli I, Huber M, Kahlert CR (Chairman of the Mother & Child Substudy), Kaiser L, Keiser O, Klimkait T, Kottanattu L, Kouyos RD, Kovari H, Kusejko K (Head of Data Centre) Ledergerber B, Martinetti G, Martinez de Tejada B, Marzolini C, Metzner KJ, Müller N, Nicca D, Paioni P, Pantaleo G, Perreau M, Polli Ch, Rauch A (Chairman of the Scientific Board), Rudin C, Schmid P, Speck R, Stöckle M (Chairman of the Clinical and Laboratory Committee), Sultan-Beyer L, Tarr P, Thanh Lecompte M, Trkola A, Vernazza P, Wagner N, Wandeler G, Weber R, Yerly S. Funding: the Swiss HIV Cohort Study has been financed within the framework of the Swiss HIV Cohort Study, supported by the Swiss National Science Foundation (grant #177499), and by the SHCS research foundation. Members of the Swiss HIV Cohort Study and the Swiss Mother and Child HIV Cohort Study:

Aebi-Popp K, Anagnostopoulos A, Battegay M, Baumann M, Bernasconi E, Böni J, Braun DL, Bucher HC, Calmy A, Cavassini M, Ciuffi A, Crisinel PA, Duppenthaler A, Dollenmaier G, Egger M, Elzi L, Fehr J, Fellay J, Francini K, Furrer H, Fux CA, Günthard HF (President of the SHCS), Haerry D (deputy of "Positive Council"), Hasse B, Hirsch HH, Hoffmann M, Hösli I, Huber M, Kahlert CR (Chairman of the Mother & Child Substudy), Kaiser L, Keiser O, Klimkait T, Kottanattu L, Kouyos RD, Kovari H, Ledergerber B, Martinetti G, Martinez de Tejada B, Marzolini C, Metzner KJ, Müller N, Nicca D, Paioni P, Pantaleo G, Perreau M, Polli Ch, Rauch A (Chairman of the Scientific Board), Rudin C, Scherrer AU (Head of Data Centre), Schmid P, Speck R, Stöckle M (Chairman of the Clinical and Laboratory Committee), Sultan-Beyer L, Tarr P, Thanh Lecompte M, Trkola A, Vernazza P, Wagner N, Wandeler G, Weber R, Yerly S. Funding: the Swiss HIV Cohort Study is supported by the Swiss National Science Foundation (grant #148522177499), and by the SHCS research foundation. **Thailand**: Program for HIV Prevention & Treatment (PHPT). Participating hospitals: Lamphun: Pornpun Wannarit; Phayao Provincial Hospital: Pornchai Techakunakorn; Chiangrai Prachanukroh: Rawiwan Hansudewechakul; Chiang Kham: Vanichaya Wanchaitanawong; Phan: Sookchai Theansavettrakul; Mae Sai: Sirisak Nanta; Prapokklao: Chaiwat Ngampiyaskul; Banglamung: Siriluk Phanomcheong; Chonburi: Suchat Hongsiriwon; Rayong: Warit Karnchanamayul; Bhuddasothorn Chacheongsao: Ratchanee Kwanchaipanich; Nakornping: Suparat Kanjanavanit; Somdej Prapinklao: Nareerat Kamonpakorn, Maneeratn Nantarukchaikul; Bhumibol Adulyadej: Prapaisri Layangool, Jutarat Mekmullica; Pranangklao: Paiboon Lucksanapisitkul, Sudarat Watanayothin; Buddhachinaraj: Narong Lertpienthum; Hat Yai: Boonyarat Warachit; Regional Health Promotion Center 6, Khon Kaen: Sansanee Hanpinitsak; Nong Khai: Sathit Potchalongsin; Samutsakhon: Pimpraphai Thanasiri, Sawitree Krikajornkitti; Phaholpolphayuhasena: Pornsawan Attavinijtrakarn; Kalasin: Sakulrat Srirojana; Nakhonpathom: Suthunya Bunjongpak; Samutprakarn: Achara Puangsombat; Mahasarakam: Sathaporn Na-Rajsima; Roi-et: Pornchai Ananpatharachai; Sanpatong: Noppadon Akarathum; Vachira Phuket: Weerasak Lawtongkum; Chiangdao: Prapawan Kheunjan, Thitiporn Suriyaboon, Airada Saipanya. Data management team: Kanchana Than-in-at, Nirattiya Jaisieng, Rapeepan Suaysod, Sanuphong Chailoet, Naritsara Naratee, and Suttipong Kawilapat. **Ukraine**: Paediatric HIV Cohort: Dr T. Kaleeva, Dr Y. Baryshnikova (Odessa Regional Centre for HIV/AIDS, Dr S. Soloha (Donetsk Regional Centre for HIV/AIDS), Dr N. Bashkatova (Mariupol AIDS Center), Dr I. Raus (Kiev City Centre for HIV/AIDS), Dr O. Glutshenko, Dr Z. Ruban (Mykolaiv Regional Centre for HIV/AIDS), Dr N. Prymak (Kryvyi Rih), Dr G. Kiseleva (Simferopol), Dr H. Bailey (UCL, London, UK). Funding acknowledgement: PENTA Foundation. **UK & Ireland**: Collaborative HIV Paediatric Study (CHIPS): Funding: CHIPS is funded by the NHS (London Specialised Commissioning Group) and has received additional support from Abbott, Boehringer Ingelheim, Bristol-Myers Squibb, GlaxoSmithKline, Gilead Sciences, Janssen and Roche. The MRC Clinical Trials Unit at UCL is supported by the Medical Research Council (https://www.mrc.ac.uk) programme number MC_UU_12023/26. CHIPS Steering Committee: Hermione Lyall (chair), Alasdair Bamford, Karina Butler, Katja Doerholt, Conor Doherty, Caroline Foster, Ian Harrison, Julia Kenny, Nigel Klein, Gillian Letting, Paddy McMaster, Fungai Murau, Edith Nsangi, , Katia Prime, Andrew Riordan, Fiona Shackley, Delane Shingadia, Sharon Storey, Gareth Tudor-Williams, Anna Turkova, Steve Welch. MRC Clinical Trials Unit: Intira Jeannie Collins, Claire Cook, Siobhan Crichton, Donna Dobson, Keith Fairbrother, Diana M. Gibb, Ali Judd, Marthe Le Prevost, Nadine Van Looy. Integrated Screening Outcome Surveillance Service (ISOSS), UCL: Helen Peters, Kate Francis, Claire Thorne. Hospitals participating in CHIPS in 2019/20: University Hospitals Birmingham NHS Foundation Trust, Birmingham: L Thrasyvoulou, S Welch; Brighton and Sussex University Hospitals NHS Trust: K Fidler; University Hospitals Bristol NHS Foundation Trust, Bristol: J Bernatoniene, F Manyika; Calderdale and Huddersfield NHS Foundation Trust, Halifax: G Sharpe; Derby Teaching Hospitals NHS Foundation Trust: B Subramaniam; Glasgow Royal Hospital for Children, Glasgow: R Hague, V Price; Great Ormond Street Hospital for Children NHS Foundation Trust, London:J Flynn, A Cardoso, M Abou – Rayyah, N Klein, A Bamford, D Shingadia; Oxford University Hospitals NHS Foundation Trust, Oxford: S Yeadon, S Segal; King's College Hospital NHS Foundation Trust, London: S Hawkins; Leeds Teaching Hospitals NHS Trust, Leeds: M Dowie; University Hospitals of Leicester NHS Trust, Leicester: S Bandi, E Percival ; Luton and Dunstable Hospital NHS Foundation Trust, Luton: M Eisenhut; K Duncan; Milton Keynes General University Hospital NHS Foundation Trust, Milton Keynes: L Anguvaa, L Wren, Newcastle upon Tyne Hospitals NHS Foundation Trust, Newcastle: T Flood, A Pickering; The Pennine Acute Hospitals NHS Trust, Manchester: P McMaster C Murphy; North Middlesex University Hospital NHS Trust, London: J Daniels, Y Lees; Northampton General Hospital NHS Trust, Northampton: F Thompson; London North West Healthcare NHS Trust, Middlesex; A Williams, B Williams, S Pope; Barts Health NHS trust, London Dr S Libeschutz; Nottingham University Hospitals NHS Trust, Nottingham: L Cliffe, S Southall; Portsmouth Hospitals NHS Trust, Portsmouth: A Freeman; Raigmore Hospital, Inverness: H Freeman; Royal Belfast Hospital for Sick Children, Belfast: S Christie; Royal Berkshire NHS Foundation Trust, Reading: A Gordon; Royal Children’s Hospital, Aberdeen: D Rosie Hague, L Clarke; Royal Edinburgh Hospital for Sick Children, Edinburgh: L Jones, L Brown; Royal Free NHS Foundation Trust, London: M Greenberg; Alder Hey Children's NHS Foundation Trust, Liverpool: C Benson, A Riordan; Sheffield Children's NHS Foundation Trust, Sheffield: L Ibberson, F Shackley; University Hospital Southampton NHS Foundation Trust, Southampton: S Patel, J Hancock; St George's University Hospitals NHS Foundation Trust, London: K Doerholt, K Prime, M Sharland, S Storey; Imperial College Healthcare NHS Trust, London: EGH Lyall, C Foster, P Seery, G Tudor-Williams, N Kirkhope, S Raghunanan; Guy's and St Thomas' NHS Foundation Trust, London: Dr Julia Kenny, A Callaghan; University Hospitals of North Midlands NHS Trust, Stoke On Trent: A Bridgwood, P McMaster; University Hospital of Wales, Cardiff: J Evans, E Blake; NHS Frimley Health Foundation Trust, Slough: A Yannoulias.

CHIPS is funded by the NHS (London Specialised Commissioning Group) and has received additional support from Bristol-Myers Squibb, Boehringer Ingelheim, GlaxoSmithKline, Roche, Abbott, and Gilead Sciences. The MRC Clinical Trials Unit at UCL is supported by the Medical Research Council (https://www.mrc.ac.uk) programme number MC_UU_12023/26. CHIPS Steering Committee: Hermione Lyall (Chair), Alasdair Bamford, Karina Butler, Katja Doerholt, Caroline Foster, Nigel Klein, Esse Menson, Paddy McMaster, Katie Prime, Andrew Riordan, Fiona Shackley, Julia Kenny, Delane Shingadia, Sharon Storey, Gareth Tudor-Williams, Anna Turkova, Steve Welch. MRC Clinical Trials Unit at UCL: Intira Jeannie Collins, Claire Cook, Siobhan Crichton, Donna Dobson, Keith Fairbrother, Diana M. Gibb, Lynda Harper, Ali Judd, Marthe Le Prevost, Nadine Van Looy. National Study of HIV in Pregnancy and Childhood: Kate Francis, Helen Peters, Claire Thorne. Participating hospitals: Republic of Ireland: Our Lady's Children’s Hospital Crumlin, Dublin: K Butler, A Walsh. UK: Birmingham Heartlands Hospital, Birmingham: L Thrasyvoulou, S Welch; Blackpool Victoria Hospital, Blackpool: N Laycock; Bristol Royal Hospital for Children, Bristol: J Bernatoniene, F Manyika ; Calderdale Royal Hospital, Halifax: G Sharpe; Coventry & Warwickshire University Hospital, Coventry: P Lewis, S Welch; Derbyshire Children’s Hospital, Derby: B Subramaniam; Derriford Hospital, Plymouth: L Hutchinson, P Ward;, Middlesex: K Sloper; Eastbourne District General Hospital, Eastbourne: K Fidler ; Glasgow Royal Hospital for Sick Children, Glasgow: R Hague, V Price; Great Ormond St Hospital for Children, London: , M Clapson, J Flynn, A Cardoso M Abou – Rayyah , N Klein, D Shingadia; Halliwell Children’s Centre, Bolton: P Ainsley-Walker; Harrogate District Hospital, Harrogate: P Tovey; Homerton University Hospital, London: D Gurtin; Huddersfield Royal Infirmary, Huddersfield: JP Garside; James Cook Hospital, Middlesbrough: A Fall; John Radcliffe Hospital, Oxford: S Yeadon , S Segal; King's College Hospital, London: C Ball, S Hawkins; Leeds General Infirmary, Leeds: M Dowie; Leicester Royal Infirmary, Leicester: S Bandi, E Percival; Luton and Dunstable Hospital, Luton: M Eisenhut; Handforth; Milton Keynes General Hospital, Milton Keynes: PK Roy; Newcastle General, Newcastle: T Flood, A Pickering; Liebeschuetz; Norfolk & Norwich Hospital, Norwich: C Kavanagh; North Manchester General, Manchester: P McMaster, C Murphy; North Middlesex Hospital, London: J Daniels, Y Lees; Northampton General Hospital, Northampton: , F Thompson; Northwick Park Hospital Middlesex; , B Williams; Nottingham QMC , Nottingham: L Cliffe, A Smyth, S Southall; Queen Alexandra Hospital, Portsmouth: H Freeman; Raigmore Hospital, Inverness: ; Royal Alexandra Hospital, Brighton: K Fidler; Royal Belfast Hospital for Sick Children, Belfast: S Christie; Royal Berkshire Hospital, Reading: A Gordon; Royal Children’s Hospital, Aberdeen: D Rogahn; Royal Cornwall Hospital, Truro: S Harris, L Hutchinson; Royal Devon and Exeter Hospital, Exeter: A Collinson, L Hutchinson; Royal Edinburgh Hospital for Sick Children, Edinburgh: L Jones, B Offerman; Royal Free Hospital, London: M Greenberg; Royal Liverpool Children’s Hospital, Liverpool: C Benson, A Riordan; Royal Preston Hospital, Preston: R O’Connor; Salisbury District General Hospital, Salisbury: N Brown; Sheffield Children's Hospital, Sheffield: L Ibberson, F Shackley; Southampton General Hospital, Southampton: SN Faust, J Hancock; St George's Hospital, London: K Doerholt, , K Prime, M Sharland, S Storey; St Luke’s Hospital, Bradford: S Gorman; St Mary’s Hospital, London: EGH Lyall, C Monrose, P Seery, G Tudor-Williams; St Thomas’ Hospital (Evelina Children’s Hospital), London: E Menson; Torbay Hospital, Torquay: J Broomhall, L Hutchinson; University Hospital Lewisham, London: D Scott, J Stroobant; Royal Stoke University Hospital, Stoke On Trent: A Bridgwood, P McMaster; University Hospital of Wales, Cardiff: J Evans, E Blake ; Wexham Park, Slough: A Yannoulias; Whipps Cross Hospital, London: M O’Callaghan.

**IeDEA CCASAnet:** Fundación Huésped, Argentina, Pedro Cahn; Universidade Federal de Minas Gerais, Brazil, Jorge Pinto; Universidade Federal de São Paulo, Brazil, Regina Célia de

Menezes Succi; Les Centres GHESKIO, Haiti, Jean William Pape; Hospital Escuela

Universitario, Honduras, Marco Tulio Luque; Instituto Hondureño de Seguridad Social,

Honduras, Denis Padgett.

**IeDEA Asia-Pacific:** National Centre for HIV/AIDS, Dermatology and STDs, Phnom Penh,

Cambodia, PS Ly, V Khol; New Hope for Cambodian Children, Phnom Penh, Cambodia, J

Tucker; Chennai Antiviral Research and Treatment Clinical Research Site (CART CRS), VHS-Infectious Diseases Medical Centre, VHS, Chennai, India, N Kumarasamy, E Chandrasekaran; BJ Medical College and Sassoon General Hospitals, Maharashtra, India, A Kinikar, V Mave, S Nimkar, I Marbaniang; Sanglah Hospital, Udayana University, Bali, Indonesia, DK Wati, D Vedaswari, IB Ramajaya; Cipto Mangunkusumo – Faculty of Medicine Universitas Indonesia, Jakarta, Indonesia, N Kurniati, D Muktiarti; Hospital Likas, Kota Kinabalu, Malaysia, SM Fong, M Lim, F Daut; Hospital Raja Perempuan Zainab II, Kelantan, Malaysia, NK Nik Yusoff, P Mohamad; Pediatric Institute, Hospital Kuala Lumpur, Kuala Lumpur, Malaysia, TJ Mohamed, MR Drawis; Penang Hospital, Penang, Malaysia, R Nallusamy, KC Chan; Department of Pediatrics, Faculty of Medicine, Chiang Mai University and Research Institute for Health Sciences, Chiang Mai, Thailand, T Sudjaritruk, V Sirisanthana, L Aurpibul; Chiangrai Prachanukroh Hospital, Chiang Rai, Thailand, R Hansudewechakul, P Ounchanum, S Denjanta, A Kongphonoi; Division of Infectious Diseases, Department of Pediatrics, Faculty of Medicine, Khon Kaen University, Khon Kaen, Thailand, P Lumbiganon, P Kosalaraksa, P Tharnprisan, T Udomphanit; PHPTIRD UMI 174 (Institut de recherche pour le développement and Chiang Mai University), Chiang Mai, Thailand, G Jourdain; HIV-NAT, The Thai Red Cross AIDS Research Centre, Bangkok, Thailand, T Puthanakit, S Anugulruengkitt, W Jantarabenjakul, R Nadsasarn; Department of Pediatrics, Faculty of Medicine Siriraj Hospital, Mahidol University, Bangkok, Thailand, K Chokephaibulkit, K Lapphra, W Phongsamart, S Sricharoenchai; Children’s Hospital 1, Ho Chi Minh City, Vietnam, KH Truong, QT Du, and CH Nguyen; Children’s Hospital 2, Ho Chi Minh City, Vietnam, VC Do, TM Ha, VT An; National Hospital of Pediatrics, Hanoi,

Vietnam, LV Nguyen, DTK Khu, AN Pham, LT Nguyen; Worldwide Orphans Foundation,

Ho Chi Minh City, Vietnam, ON Le; TREAT Asia/amfAR -- The Foundation for AIDS Research,

Bangkok, Thailand, AH Sohn, JL Ross, T Suwanlerk; The Kirby Institute, UNSW Australia,

Sydney, Australia, MG Law, A Kariminia.

**IeDEA Central Africa:** *Site investigators and cohorts:* Nimbona Pélagie, ANSS, Burundi; Patrick Gateretse, Jeanine Munezero, Valentin Nitereka, Théodore Niyongabo, Christelle Twizere, Centre National de Reference en Matiere de VIH/SIDA, Burundi; Hélène Bukuru, Thierry Nahimana, CHUK, Burundi; Jérémie Biziragusenyuka, Risase Scholastique Manyundo, HPRC, Burundi; Tabeyang Mbuh, Kinge Thompson Njie, Edmond Tchassem, Kien-Atsu Tsi, Bamenda Hospital, Cameroon; Rogers Ajeh, Mark Benwi, Anastase Dzudie, Akindeh Mbuh, Marc Lionel Ngamani, Victorine Nkome, CRENC & Douala General Hospital, Cameroon; Djenabou Amadou, Eric Ngassam, Eric Walter Pefura Yone, Jamot Hospital, Cameroon; Alice Ndelle Ewanoge, Norbert Fuhngwa, Chris Moki, Denis Nsame Nforniwe, Limbe Regional Hospital, Cameroon; Catherine Akele, Faustin Kitetele, Patricia Lelo, Martine Tabala, Kalembelembe Pediatric Hospital, Democratic Republic of Congo; Emile Wemakoy Okitolonda, Landry Wenzi, Kinshasa School of Public Health, Democratic Republic of Congo; Merlin Diafouka, Martin Herbas Ekat, Dominique Mahambou Nsonde, CTA Brazzaville, Republic of Congo; Adolphe Mafou, CTA Pointe-Noire, Republic of Congo; Fidele Ntarambirwa, Bethsaida Hospital, Rwanda; Yvonne Tuyishimire, Busanza Health Center, Rwanda; Theogene Hakizimana, Gahanga Health Center, Rwanda; Josephine Ayinkamiye, Gikondo Health Center, Rwanda; Sandrine Mukantwali, Kabuga Health Center, Rwanda; Henriette Kayitesi, Olive Uwamahoro, Kicukiro Health Center, Rwanda; Viateur Habumuremyi, Jules Ndumuhire, Masaka Health Center, Rwanda; Joyce Mukamana, Yvette Ndoli, Oliver Uwamahoro, Nyarugunga Health Center, Rwanda; Gallican Kubwimana, Pacifique Mugenzi, Benjamin Muhoza, Athanase Munyaneza, Emmanuel Ndahiro, Diane Nyiransabimana, Jean d'Amour Sinayobye, Vincent Sugira, Rwanda Military Hospital, Rwanda; Chantal Benekigeri, Gilbert Mbaraga, WE-ACTx Health Center, Rwanda. *Coordinating and Data Centers:* Adebola Adedimeji, Kathryn Anastos, Madeline Dilorenzo, Lynn Murchison, Jonathan Ross, Albert Einstein College of Medicine, USA; Diane Addison, Margaret Baker, Ellen Brazier, Heidi Jones, Elizabeth Kelvin, Sarah Kulkarni, Grace Liu, Denis Nash, Matthew Romo, Olga Tymejczyk, Institute for Implementation Science in Population Health, Graduate School of Public Health and Health Policy, City University of New York (CUNY), USA; Batya Elul, Columbia University, USA; Xiatao Cai, Don Hoover, Hae-Young Kim, Chunshan Li, Qiuhu Shi, Data Solutions, USA; Robert Agler, Kathryn Lancaster, Marcel Yotebieng, Ohio State University, USA; Mark Kuniholm, University at Albany, State University of New York, USA; Andrew Edmonds, Angela Parcesepe, University of North Carolina at Chapel Hill, USA; Olivia Keiser, University of Geneva; Stephany Duda; Vanderbilt University School of Medicine.

**IeDEA East Africa:** Lameck Diero, Samuel Ayaya (Academic Model Providing Access to Healthcare (AMPATH), Eldoret, Kenya), Elizabeth Bukusi (Family Family AIDS Care & Education Services (FACES), Kisumu, Kenya), Charles Kasozi (Masaka Regional Referral Hospital, Masaka, Uganda), Mwebesa Bosco Bwana (Mbarara University of Science and Technology (MUST), Mbarara, Uganda), Barbara Castelnuovo (Infectious Diseases Institute (IDI), Kampala, Uganda), Fred Nalugoda (Rakai Health Sciences Program (RHSP), Kalisizo, Uganda), Geoffrey Somi (National AIDS Control Program (NACP) Domoda, Tanzania), and Mark Urassa (National Institute for Medical Research (NIMR), Mwanza, Tanzania), Batya Elul (Columbia University, New York, USA), Rachel Vreeman (Mt. Sinai, New York, USA) Jennifer Syvertsen (University of California Riverside, California, USA) Rami Kantor (Brown University, Providence, USA), Jeff Martin (University of California, San Francisco, USA), Craig Cohen (University of California, San Francisco, USA), Paula Braitstein (University of Toronto, Toronto, Canada) East Africa IeDEA Regional Data Center, Indiana University: Kara Wools-Kaloustian, Constantin Yiannoutsos, and Beverly Musick.

**IeDEA Southern Africa:** Aid for AIDS, South Africa, Gary Maartens; Aurum Institute for Health

Research, South Africa, Christopher J. Hoffmann; Centre for Infectious Disease Research in

Zambia, Zambia, Michael Vinikoor; Centro de Investigaçao em Saude de Manhiça, Mozambique, Eusebio Maceta; Dignitas, Malawi, Monique van Lettow; Gugulethu Cohort (Desmond Tutu HIV Centre), South Africa, Robin Wood; Harriet Shezi Clinic, Chris Hani Baragwanath Hospital (Wits Paediatric HIV Clinics), South Africa, Shobna Sawry; Hlabisa (Africa Centre for Health & Population Studies), South Africa, Frank Tanser; Khayelitsha ART Programme, South Africa, Andrew Boulle; Kheth ‘Impilo, South Africa, Geoffrey Fatti; Lighthouse Truse clinic, Malawi, Sam Phiri; McCord Hospital, South Africa, Janet Giddy; Newlands Clinic, Zimbabwe, Cleophas Chimbetete; Queen Elizabeth Hospital, Malawi, Kennedy Malisita; Rahima Moosa Mother & Child Hospital (Wits Paediatric HIV Clinics), South Africa, Karl Technau; Red Cross War Memorial Children’s Hospital and School of Child & Adolescent Health, University of Cape Town, South Africa, Brian Eley; SolidarMed SMART Programme Lesotho, Lesotho, Christiane Fritz; SolidarMed SMART Programme Mozambique, Mozambique, Michael Hobbins; SolidarMed SMART Programme Zimbabwe, Zimbabwe, Kamelia Kamenova; Themba Lethu Clinic, Helen Joseph Hospital, South Africa, Matthew P. Fox; Tygerberg Academic Hospital, South Africa, Hans Prozesky.

**IeDEA West Africa:** Executive Committee: François Dabis (Principal Investigator, Bordeaux,

France), Emmanuel Bissagnene (Co-Principal Investigator, Abidjan, Côte d’Ivoire), Elise Arrivé

(Bordeaux, France), Patrick Coffie (Abidjan, Côte d’Ivoire), Didier Ekouevi (Abidjan, Côte d’Ivoire), Antoine Jaquet (Bordeaux, France), Valériane Leroy (Chair of the pediatric group,

Toulouse, France). Benin, Cotonou: Sikiratou Koumakpaï, (CNHU Hubert Maga).Côte d’Ivoire, Abidjan: Marie-Sylvie N’Gbeche, Kouadio Kouakou (CIRBA); Madeleine Amorissani Folquet

(CHU Cocody); Tanoh François Eboua (CHU Yopougon). Ghana, Accra: Lorna Renner (Korle

Bu TH). Mali, Bamako: Fatoumata Dicko, Mariam Sylla (CH Gabriel Toure). Togo, Lomé: Elom

Takassi (CHU Tokoin/Sylvanus Olympio). Senegal, Dakar: Haby Signate-Sy, Hélène Dior (CH

Albert Royer).Burkina Faso, Ouagadougou: Diarra Yé, Fla Kouéta (CH Charles de Gaulle).

**IMPAACT:** We thank the children and families for their participation in PACTG 219C and IMPAACT P1074, and the individuals and institutions involved in the conduct of these protocols as well as the leadership and participants of the respective protocol teams. We are grateful for the contributions of Joyce Kraimer, Barbara Heckman, Shirley Traite, Miriam Chernoff, and Nathan Tryon. *Protocol 219C and IMPAACT P1074 Team Members:* Russell Van Dyke MD, Chair; James M Oleske MD MPH, Founding Chair; Mark Abzug MD and John Farley MD, Vice-Chairs; Mary Glen Fowler MD MPH, Michael Brady MD and Wayne Dankner MD, Past Vice-Chairs; Elizabeth Smith MD, Anne Fresia, Gregory Ciupak, Michelle Eagle PA, Dorothy R Smith MS CPNP, Paul Palumbo MD, John Sleasman MD, James Connor MD, Michael Hughes PhD, Rebecca Oyomopita MSc, George Johnson MD, Andrew Wiznia MD, Nancy Hutton MD, Andrea Kovacs MD, Mary Sawyer MD, Martin Anderson MD, Audrey Rogers PhD MPH, William Borkowsky MD, Jane Lindsey ScD, Jack Moye MD, Myron Levin MD, Marilyn Crain MD MPH, Paul Britto MS, Ruth Toumala MD, Joseph Cervia MD, Eileen Monagham, Kenneth Dominguez MD, Melody Higgins RN MS, George Seage DSc MPH, Denise Gaughan MPH, Phil Gona PhD, William Shearer MD PhD, Lois Howland DPH MS RN, Deborah Storm PhD RN, Kathleen Malee PhD, Wendy Mitchell MD, Carol Gore, Eve Powell, Michelle McConnell MD, Newana Beatty, Susan Brogly PhD, Jennifer Bryant CRA, Miriam Chernoff PhD, Barbara Heckman BS, Dawn English, Edward Handelsman MD, Patrick Jean-Philippe MD, Kathleen Kaiser, Joyce Kraimer MS , Linda Millar, Shirley Traite MSW, Paige Williams PhD, Elizabeth Woods MD MPH, Carol Worrell MD.

The following sites participated in PACTG 219/219C: University of New Jersey Medical and Dental School - Department of Pediatrics, Division of Allergy, Immunology & Infectious Diseases,Boston Medical Center, Division of Pediatric Infectious Diseases, Med, Children’s Hospital LA - Department of Pediatrics, Division of Clinical Immunology & Allergy, Long Beach Memorial Medical Center, Miller Children's Hospital, Harbor - UCLA Medical Center - Department of Pediatrics, Division of Infectious Diseases, Johns Hopkins Hospital & Health System - Department of Pediatrics, Division of Infectious Diseases, University of Maryland Medical Center, Division of Pediatric Immunology & Rheumatology, Texas Children's Hospital, Allergy & Immunology Clinic, Cook County Hospital, Children's Hospital of Columbus, Ohio, University of Miami Miller School of Medicine, Division of Pediatric Immunology & Infectious Disease, University of California San Francisco School of Medicine, Department of Pediatrics, Children's Hospital & Research Center Oakland, Pediatric Clinical Research Center & Research Lab, University of California San Diego Mother, Child & Adolescent HIV Program, Duke University School of Medicine - Department of Pediatrics, Children's Health Center, University of North Carolina at Chapel Hill School of Medicine - Department of Pediatrics, Division of Immunology and Infectious Diseases, Schneider Children’s Hospital, Harlem Hospital Center, New York University School of Medicine, Division of Pediatric Infectious Diseases, Children's National Medical Center, ACT, University of Washington School of Medicine - Children's Hospital and Regional Medical Center, University of Illinois College of Medicine at Chicago, Department of Pediatrics, Yale University School of Medicine - Department of Pediatrics, Division of Infectious Disease, SUNY at Stony Brook School of Medicine, Division of Pediatric Infectious Diseases, Howard University Hospital, Department of Pediatrics & Child Health, LA County/University of Southern California Medical Center, University of Florida Health Science Center Jacksonville, Division of Pediatric Infectious Disease & Immunology, North Broward Hospital District, Children's Diagnostic & Treatment Center, University of Rochester Medical Center, Golisano Children's Hospital, Medical College of Virginia, St. Jude Children's Research Hospital, Department of Infectious Diseases, University of Puerto Rico, U. Children’s Hospital AIDS, Children's Hospital of Philadelphia, Center for Pediatric & Adolescent AIDS, St. Christopher’s Hospital for Children/Drexel University College of Medicine, Bronx-Lebanon Hospital Center, Infectious Diseases, New York Medical College/Metropolitan Hospital Center, University of Massachusetts Memorial Children's Medical School, Department of Pediatrics, Baystate Health, Baystate Medical Center, Connecticut Children's Medical Center, Medical College of Georgia School of Medicine, Department of Pediatrics, Division of Infectious Disease, University of South Alabama College of Medicine, Southeast Pediatric ACTU, LSU Health Sciences Center, Tulane University Health Sciences Center, St. Josephs Hospital and Medical Center, Cooper University Hospital - Children's Hospital Boston, Division of Infectious Diseases, David Geffen School of Medicine at UCLA - Department of Pediatrics, Division of Infectious Diseases, Children's Hospital of Orange County, Children's Memorial Hospital - Department of Pediatrics, Division of Infectious Disease, University of Chicago - Department of Pediatrics, Division of Infectious Disease, Mt. Sinai Hospital Medical Center - Chicago, Women’s & Children’s HIV Program, Columbia University Medical Center, Pediatric ACTU, Incarnation Children’s Center, Cornell University, Division of Pediatric Infectious Diseases & Immunology, University of Miami Miller School of Medicine - Jackson Memorial Hospital, Bellevue Hospital (Pediatric), San Francisco General (Pediatric), Phoenix Children's Hospital, Metropolitan Hospital Center (N.Y.), University of Cincinnati, SUNY Downstate Medical Center, Children's Hospital at Downstate, North Shore University Hospital, Jacobi Medical Center, University of South Florida - Department of Pediatrics, Division of Infectious Diseases, Cornell University, Oregon Health & Science University - Department of Pediatrics, Division of Infectious Diseases, Children's Hospital of the King's Daughters, Infectious Disease, Lincoln Medical & Mental Health Center, Mt. Sinai School of Medicine, Division of Pediatric Infectious Diseases, Emory University Hospital, San Juan City Hospital, UMDNJ - Robert Wood Johnson, Ramon Ruiz Arnau University Hospital, Medical University of South Carolina, SUNY Upstate Medical University, Department of Pediatrics, Wayne State University School of Medicine, Children's Hospital of Michigan, Children’s Hospital at Albany Medical Center, Children’s Medical Center of Dallas, Children's Hospital - University of Colorado at Denver and Health Sciences, Center, Pediatric Infectious Diseases, Columbus Children’s Hospital, University of Florida College of Medicine - Department of Pediatrics, Division of Immunology, Infectious Diseases & Allergy, University of Mississippi Medical Center, Palm Beach County Health Department, Children’s Hospital LA - Department of Pediatrics, Division of Adolescent Medicine, Vanderbilt University Medical Center, Division of Pediatric Infectious Diseases, Washington University School of Medicine at St. Louis, St. Louis Children's Hospital, Children’s Hospital & Medical Center, Seattle ACTU, Oregon Health Sciences University, St. Luke's-Roosevelt Hospital Center, Montefiore Medical Center - Albert Einstein College of Medicine, Children's Hospital, Washington, D.C., Children’s Hospital of the King's Daughters, University of Alabama at Birmingham, Department of Pediatrics, Division of Infectious Diseases, Columbus Regional HealthCare System, The Medical Center, Sacred Heart Children’s Hospital/CMS of Florida, Bronx Municipal Hospital Center/Jacobi Medical Center.

The following sites participated in P1074: New Jersey Medical School, UCLA–Los Angeles/Brazil AIDS Consortium, Texas Children’s Hospital, Lurie Children’s Hospital of Chicago, Columbia University Medical Center, University of Miami Pediatric Perinatal HIV/AIDS, University of California San Diego, Mother-Child-Adolescent Program, Duke University Medical Center, Children’s Hospital of Boston, Boston Medical Center Pediatric HIV Program, New York University, Jacobi Medical Center Bronx, Children’s National Medical Center Washington, DC, Seattle Children’s Hospital, University of South Florida Tampa, San Juan City Hospital, SUNY Stony Brook, Children’s Hospital of Michigan, Howard University Washington DC, Harbor UCLA Medical Center, University of Southern California School of Medicine, University of Florida Health Science Center, University of Colorado Denver, South Florida Children’s Diagnostic and Treatment Center Fort Lauderdale, Strong Memorial Hospital University of Rochester Medical Center, Rush University Cook County Hospital Chicago, Children’s Hospital of Los Angeles, University of California San Francisco, Johns Hopkins University Baltimore, Miller Children’s Hospital, University of Maryland Baltimore, Tulane University New Orleans, University of Alabama Birmingham, The Children’s Hospital of Philadelphia, Bronx-Lebanon Hospital, St Jude’s Children’s Hospital, University of Puerto Rico Pediatric HIV/AIDS Research Program, Western New England Maternal Pediatric Adolescent AIDS.

**PHACS:** The following sites participated in PHACS AMP: Ann & Robert H. Lurie Children’s Hospital of Chicago: Ram Yogev; Baylor College of Medicine: William Shearer; Bronx Lebanon

Hospital Center: Murli Purswani; Children's Diagnostic & Treatment Center: Ana Puga; Children’s Hospital, Boston: Sandra K. Burchett; Jacobi Medical Center: Andrew Wiznia; Rutgers – New Jersey Medical School: Arry Dieudonne; St. Christopher’s Hospital for Children: Janet S. Chen; St. Jude Children's Research Hospital: Katherine Knapp; San Juan Hospital/Department of Pediatrics: Midnela Acevedo-Flores; Tulane University School of Medicine: Margarita Silio; University of California, San Diego: Stephen A. Spector; University of Colorado Denver Health Sciences Center: Elizabeth McFarland; University of Miami: Gwendolyn Scott. Project coordination was provided by Harvard T.H. Chan School of Public Health: Julie Alperen and by Tulane University School of Medicine: Patrick Davis. Data management was provided by Frontier Science and Technology Research Foundation: Sue Siminski; Operational and regulatory support was provided by Westat Inc: Julie Davidson.

**Optimal Models-ICAP:** The following people and sites contributed to Optimal Models: ***Ethiopia***

-Mohamed Ahmed, Harari Regional Health Bureau; Zelalem Habtamu, Oromia Regional Health

Bureau; Kassahun Hailegiorgis, Dire Dawa Regional Health Bureau; Zenebe Melaku, ICAP Ethiopia. Abomsa Hospital, Abosto HC, Adama Hospital, Addis Ketema Health Center, Adola, Ambo Hospital, Arategna Health Center, Assela Hospital, Bishoftu Hospital, Bisidimo Hospital, Bulle Hura Hospital, Chiro Hospital, Deder Hospital, Dire Dawa Health Center, Dodola, Fitche Hospital, Gedo Hospital, Gelemso Hospital, Gende Gerada Health Center, Gende Kore Health Center, Gindeberet Hospital, Ginir Hospital, Goro Health Center, Harar TB Hospital, Hiwot Fana Hospital, Jenila Health Center, Jijiga Health Center, Jimma Hospital, Karamara Hospital, Kuyu Hospital, Leghare Health Center, Limmu-Genet Hospital, Mariam Work Hospital, Melka-Jebdu Health Center, Metehara Hospital, Metu Karl Hospital, Misrak Arbegnoch Hospital, Negele Hospital, Robe Dida, Sabian Health Center, Shashemene Hospital, Sher Ethiopia Private Hospital, St. Luke (Wolisso) Hospital, Tulu Bolo Hospital, Wonji Hospital, Yabelo, Yimaji Private Hospital. ***Kenya*** - Mark Hawken, ICAP Kenya; Maureen Kamene Kimenye, PASCO Central province; Irene N. Mukui, National AIDS and STIs Control Programme. Abidha Health Center, Ahero Sub District Hospital, Akala Health Center, Aluor Mission, Athi River Health Center, Awasi Mission, Bar Agulu Dispensary, Bar Olengo, Bondo District Hospital, Boro Dispensary, Daniel Comboni Dispensary-Ndithini, Dienya Health Center, Gobei Dispensary, Hawinga Dispensary, Kali Dispensary, Kathiani Sub District Hospital, Kibwezi Health Center, Kikoko Mission Hospital, Kitui District hospital, Madiant District Hospital, Mahaya Health Center, Malanga Health Center, Manyuanda Dispensary, Masogo Subdistrict Hospital, Matangwe mission Hospital, Mtito Andei Health Center, Muhoroni SDH, Mulaha Dispensary, Naya Dispensary, Ndere Health Center, Ndori Dispensary, Ngiya Mission, Nyakach AIC Dispensary, Nyangoma Kogelo, Ongielo Health Center, Rangala Mission, Rera Health Center, Siaya District Hospital, Sigomere Health Center, Sikalame Health Center, Tawa Health Center, Tingwangi Health Center, Ukwala Health Center, Usigu Health Center, Uyawi Health Center, Wagai Dispensary, Yala Sub District Hospital. ***Mozambique*** - Josue Lima, ICAP Mozambique; Antonio Mussa, ICAP Mozambique; Américo Rafi Assan, Ministry of Health Mozambique. 17 de Setembro Health Center, 25 de Setembro Health Center – Nampula, Akumi Health Center – Nacala, Anchilo Health Center, Angoche HR, Coalane Health Center, Gurue Rural Hospital Ilha de Mocambique, Liupo Health Center, Lumbo Health Center, Malema Health Center, Marrere General Hospital, Meconta Health Center, Memba Health Center, Milange Health Center, Military Hospital-Maputo, Mocuba Rural Hospital, Moma Health Center, Monapo HR, Monapo Health Center, Mossuril HR, Nacala-Porto District Hospital, Nacala-Porto Health Center, Nacuxa HR, Nametil Health Center, Namialo Health Center, Namitoria Health Center, Nampula Central Hospital, Namuinho Health Center, Nicoadala Health Center, Pediatric Central Hospital – Nampula, Ribaue Rural Hospital; ***Rwanda*** - Vincent Mutabazi, Treatment and Research AIDS Center; Ruben Sahabo, ICAP Rwanda. Avega Clinic, Bethsaida Health Center, Bigogwe Health Center, Busasamana Health Center, Butare Hospital, Carrefour Polyclinic, Central Hospital- Kigali, Congo Nil Health Center, Gisenyi District Hospital, Gisenyi Prison, Gisovu Health Center, Kabaya District Hospital, Kabusunzu Health Center, Kayove Health Center, Kibuye District Hospital, Kicukiro Health Center, Kigali Central Prison, Kigufi Health Center, Kinunu Health Center, Kirambo Health Center, Kirinda District Hospital, Kivumu Health Center, Mugonero District Hospital, Muhima District Hospital, Muhororo District Hospital, Mukungu Health Center, Munzanga Health Center, Murunda District Hospital, Mushubati Health Center, Mwendo Health Center, Ndera Neuropsychiatric Hospital, Nyabirasi Health Center, Nyakiriba Health Center, Nyange A health Center, Nyange B Health Center, Ramba Health Center, Rambura Health Center, Rubengera Health Center, Rugarama Health Center, Rususa health Center, Shyira

District Hospital; ***Tanzania*** - Gretchen Antelman, ICAP Tanzania; Redempta Mbatia, ICAP Tanzania; Geoffrey Somi, National AIDS Control Program. Al-Rahma Hospital, Bagamoyo District Hospital, Baleni Dispensary, Biharamulo Designated District Hospital, Bunazi Health Center, Bwanga Health Center, Chake Chake Hospital, Chalinze Health Center, Chato District Hospital, ChemChem (Miburani) Dispensary, Heri Mission Hospital, Ikwiriri Health Center, Isingiro Hospital, Izimbya Hospital, Kabanga Mission Hospital, Kagera Sugar Hospital, Kagondo

Hospital, Kahororo Dispensary, Kaigara Health Center, Kakonko Health Center, Kanazi Health Centre, Kasulu District Hospital, Katoro Health Centre, Kayanga Health Centre, Kibiti Health Center, Kibondo District Hospital, Kigarama Health Centre, Kigoma Dispensary, Kigoma Regional Hospital, Kilimahewa Mission Dispensary, Kirongwe Dispensary, Kisarawe District Hospital, Kishanje Health Centre, Kisiju Health Centre, Kivunge Hospital, Kongowe Dispensary,

Lugoba Health Center, Mabamba Health Center, Mafia District Hospital, Maneromango Health Center, Masaki Health Centre, Mchukwi Hospital, Michiweni Hospital, Miono Health Centre, Mkamba Health Centre, Mkoani Health Centre, Mkomaindo Hospital, Mkuranga District Hospital, Mlandizi Health Center, Mnazi Mmoja Hospital, Mugana Designated District Hospital, Murgwanza Designated District Hospital, Murongo Health Center, Mwembeladu Maternity Hospital, Mzenga Health Centre, Ndanda Hospital, Ndolage Hospital, Newala Hospital, Nguruka

Health Centre, Nkwenda Health Center, Nyakahanga Designated District Hospital, Nyamiaga Health Centre, Ocean Road Cancer Institute, Rubya Designated District Hospital, Rulenge Hospital, Rwamishenye Health Centre, St. Therese Bukoba Health Center, Tumbi Regional Hospital, Ujiji Health Center, Utende Dispensary, Utete District Hospital, Uvinza Dispensary, Wete Hospital, Zam Zam Health Centre. ***ICAP Central*** - Matthew Lamb, Denis Nash, Harriet Nuwagaba-Biribonwoha, Chloe Teasdale.
